# Boosting electrocatalytic performance via electronic structure regulation for acidic oxygen evolution

**DOI:** 10.1016/j.isci.2023.108738

**Published:** 2023-12-18

**Authors:** Qian Wu, Qingping Gao, Xingpeng Wang, Yuping Qi, Li Shen, Xishi Tai, Fan Yang, Xun He, Yan Wang, Yongchao Yao, Yuchun Ren, Yonglan Luo, Shengjun Sun, Dongdong Zheng, Qian Liu, Sulaiman Alfaifi, Xuping Sun, Bo Tang

**Affiliations:** 1 Department of Chemistry and Chemical Engineering, Weifang University, Weifang 261061, Shandong, China; 2 Department of Chemical Engineering, Weifang Vocational College, Weifang 262737, Shandong, China; 3 Institute of Fundamental and Frontier Sciences, University of Electronic Science and Technology of China, Chengdu 610054, Sichuan, China; 4 College of Chemistry, Chemical Engineering and Materials Science, Shandong Normal University, Jinan 250014, Shandong, China; 5 Institute for Advanced Study, Chengdu University, Chengdu 610068, Sichuan, China; 6 Chemistry Department, Faculty of Science, King Abdulaziz University, Jeddah 21589, Saudi Arabia; 7 Laoshan Laboratory, Qingdao 266237, Shandong, China

**Keywords:** Physical chemistry, Materials chemistry, Energy materials

## Abstract

High-purity hydrogen produced by water electrolysis has become a sustainable energy carrier. Due to the corrosive environments and strong oxidizing working conditions, the main challenge faced by acidic water oxidation is the decrease in the activity and stability of anodic electrocatalysts. To address this issue, efficient strategies have been developed to design electrocatalysts toward acidic OER with excellent intrinsic performance. Electronic structure modification achieved through defect engineering, doping, alloying, atomic arrangement, surface reconstruction, and constructing metal-support interactions provides an effective means to boost OER. Based on introducing OER mechanism commonly present in acidic environments, this review comprehensively summarizes the effective strategies for regulating the electronic structure to boost the activity and stability of catalytic materials. Finally, several promising research directions are discussed to inspire the design and synthesis of high-performance acidic OER electrocatalysts.

## Graphical abstract

**Figure:**
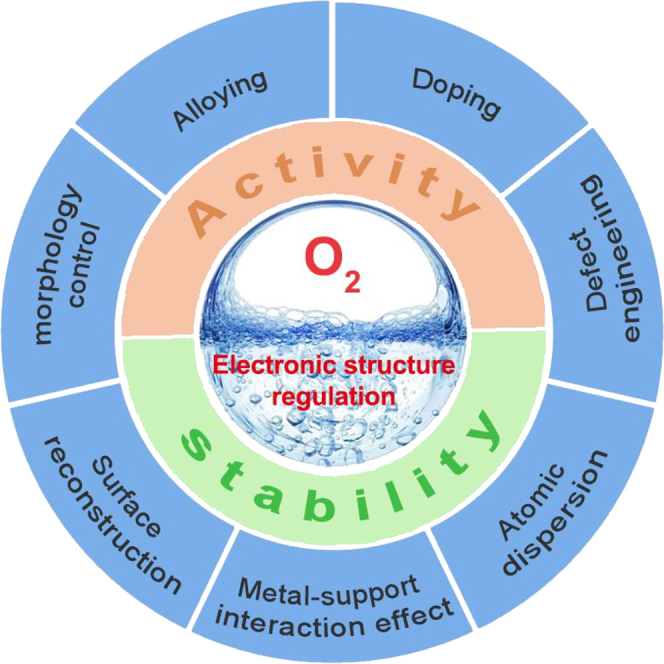

## Introduction

Hydrogen energy, as a promising renewable and clean energy source, has attracted global attention and intensive investigations.^1^^,^^2^^,^^3^^,^^4^^,^^5^ Compared with other fuels, hydrogen has a very high mass energy density of 142 MJ/kg, which is about 3 times that of traditional petroleum and 4.5 times that of coal.^6^^,^^7^^,^^8^ In addition, effective utilization of hydrogen energy is expected to achieve zero or near zero emissions of pollutants.^9^^,^^10^^,^^11^^,^^12^ Several approaches of hydrogen production have been widely used, including steam methane reforming, natural gas and coal gasification.^13^^,^^14^^,^^15^ However, these hydrogen production processes are usually accompanied by the consumption of fossil fuels and high concentration of CO_2_ emission, which are not conducive to the development of long-term green economy. By contrast, water electrolysis driven by renewable energy (such as wind and solar) and abundant water resources, is more appealing toward green hydrogen and decarbonized future.^16^^,^^17^^,^^18^ To date, water electrolysis is largely challenged by the anodic sluggish oxygen evolution reaction (OER), which is suffered from the proton-coupled multi-electron transfer steps in both acidic and alkaline environments.^19^^,^^20^^,^^21^ In contrast to traditional alkaline electrolyzers, proton exchange membrane water electrolysis (PEMWE) offers a much higher conductivity, faster response and lower ohmic losses with less gas crossover, and has been recognized as the most suitable candidate for coupling with fluctuant energy input.^22^^,^^23^^,^^24^ Nevertheless, the harsh acid-corrosive condition largely restricts the catalytic materials selection for PEMWE, especially for its OER electrocatalysts.^25^^,^^26^ Currently, only a few more stable noble metal materials, such as Ir and Ru, can be compatible with PEM, but unfortunately, the low natural abundance and high price of these materials limit the future popularization of the application. Unsurprisingly, introducing low-cost non-precious metals to partially replace noble metals to design and synthesize catalytic materials with low Ir or Ru content has become the main preparation strategy for most emerging precious metal-based electrocatalysts.^27^ In addition, numerous studies have reported that some noble-metal-free materials exhibited potential activity and durability for acidic OER.^28^^,^^29^^,^^30^^,^^31^^,^^32^^,^^33^ Unfortunately, most of these catalysts are unable to achieve durable performance under harsh acidic OER operating conditions. Therefore, the development of acid-resistant OER catalysts with high efficiency is urgent, yet challenging.

Compared to hydrogen evolution reaction (HER) with dual-electron transfer, OER process accompanied by four-proton-electron coupling requires a higher overpotential to overcome the relatively sluggish reaction kinetics.^19^^,^^20^ Therefore, in this sense, OER governs the overall efficiency of water electrolysis. The adsorption strength of the reaction intermediates on the active sites of catalysts determines the kinetic activation barrier of OER. Based on Sabatier’s principle, an ideal catalyst should have a moderate intermediate binding energy.^34^ Notably, the adsorption strength of intermediates is closely related to the electronic structure of catalyst.^35^ According to the density functional theory (DFT) calculations, the increased density of state (DOS) of active sites near the Fermi level can upshifting the d-band center, which reduces the electron filling by coupling with the O 2p orbits to form the higher anti-bond orbit, thereby modulating the adsorption strength of key intermediates and ultimately leading to the reduced energy barrier of the rate-determining step (RDS).^36^^,^^37^^,^^38^ Furthermore, many studies have showed that the construction of metal-oxygen covalent bonds can optimize the adsorption for reaction intermediates, and eventually enhance the OER catalytic activity.^39^^,^^40^^,^^41^ Consequently, modulation of the electronic structure of catalysts have viewed as a promising strategy for promoting the acidic OER.

**Figure 1:**
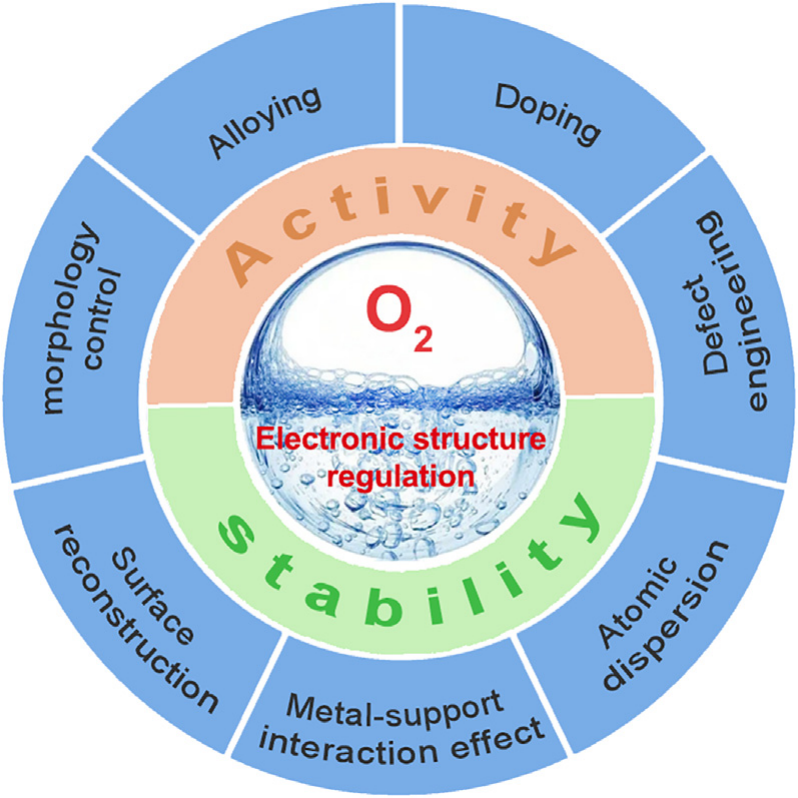
Schematic illustration of various electronic structure strategies for efficient OER electrocatalysts in acidic environment.

Numerous research results have shown that the electronic structure of catalysts can be regulated through various strategies, such as doping engineering, alloying, atomic dispersion, and morphology control. Therefore, a large number of OER electrocatalysts harvesting high performance through electronic structure modulation in acidic media have emerged in recent years. Rather than discussing these catalysts in detail, this mini-review primarily focuses on the effective strategies for rationally designing the desired OER electrocatalysts toward high-performance acidic water oxidation. We first give a detailed discussion of the reaction mechanisms during the OER process, which are key to understanding and guiding the design of effective electrocatalysts. Based on summarizing the reasonable catalyst design strategies, the current challenges of developing efficient OER electrocatalysts and the future perspectives of designing high-performance PEM water electrolyzers are finally highlighted (Figure 1).

## Reaction mechanisms

To design and synthesize efficient electrocatalysts with optimal activity and stability, various possible mechanisms have been proposed based on kinetic and/or thermodynamic analysis to describe and explain the OER process in acidic medium.^42^^,^^43^ Among them, the most widely accepted are the traditional adsorbate evolution mechanism (AEM) and lattice oxygen evolution mechanism (LOM), which differ essentially in the origin of the generated oxygen atoms.^35^ In AEM, all the generated oxygen comes from the oxygenated intermediates in water, whereas in LOM, it partially or entirely originates from the lattice oxygen of electrocatalyst itself.^44^^,^^45^ Research has shown that the crystallinity of catalytic materials typically has a significant impact on the specific reaction mechanism followed by OER catalytic process.^46^ Generally, well-crystallinited oxides with few defects tend to employ AEM to catalyze the OER process, while the amorphous metal oxides with abundant oxygen vacancies and some chalcogenides with high metal-oxygen covalency tend to control the reaction pathway by LOM.^46^

### Adsorbate evolution mechanism

**Figure 2:**
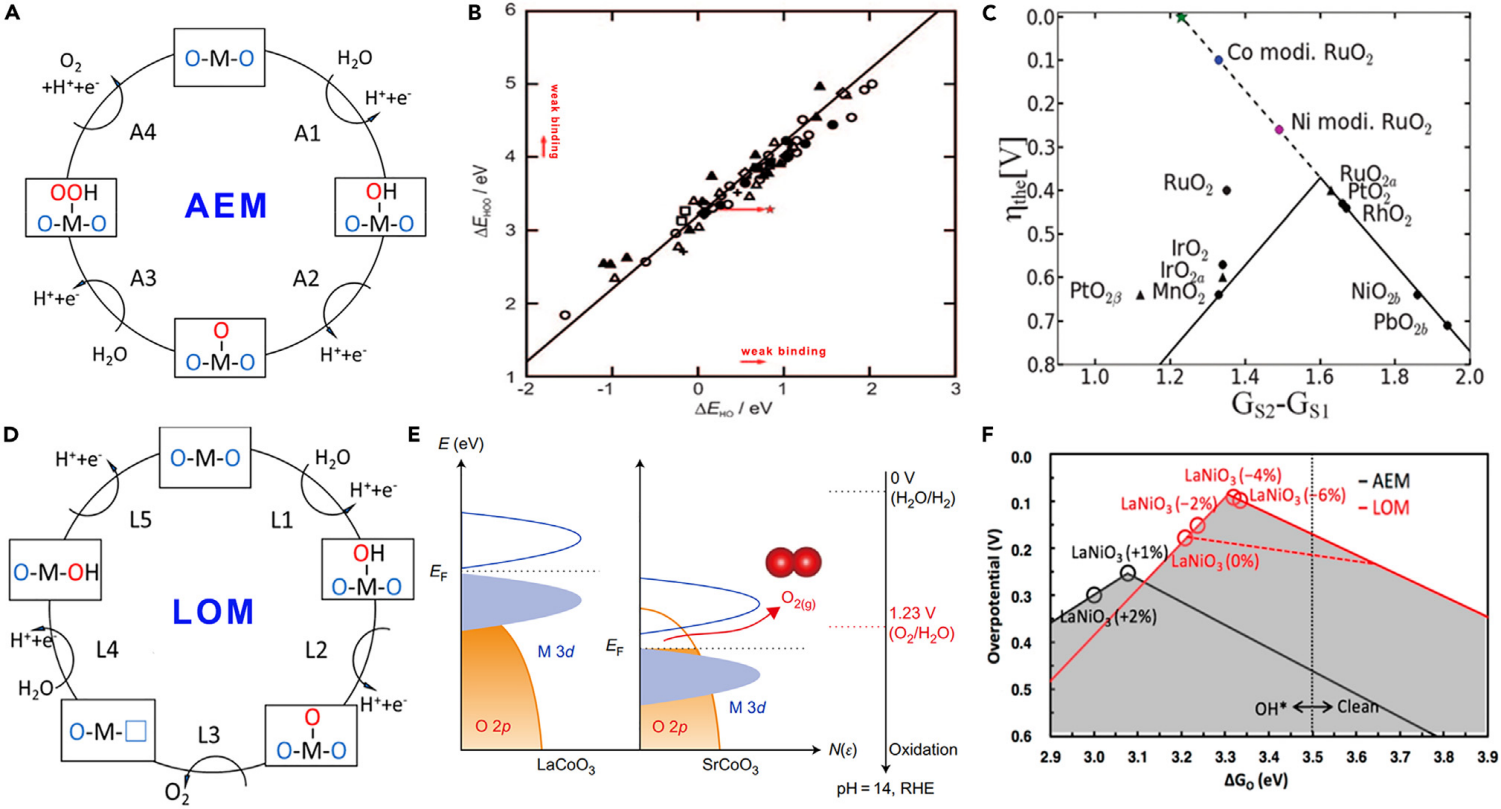
Mechanistic understanding of OER in acidic environment (A) Detailed electron transfer pathway of the adsorbate evolution mechanism. Reproduced with permission from (Shan et al., 2019). Copyright @ 2019 American Chemical Society. (B) Adsorption energy of HOO∗ plotted against the adsorption energy of HO∗ on different oxides. Reproduced with permission from (Man et al., 2011). Copyright @ 2011 Wiley-VCH Verlag GmbH. (C) Volcano curve of theoretical overpotential for OER using the second charge-transfer reaction as a descriptor. Reproduced with permission from (Halck et al., 2014). Copyright @ 2014 Royal Society of Chemistry. (D) Lattice oxygen participation mechanism pathway. Reproduced with permission from (Shan et al., 2019). Copyright @ 2019 American Chemical Society. (E) Scheme of rigid band diagrams for LaCoO_3_ and SrCoO_3_. Reproduced with permission from (Grimaud et al., 2017). Copyright @ 2017 Nature Publishing Group. (F) Overall OER volcano that considers both AEM (black) and LOM (red) for perovskites. Reproduced with permission from (Yoo et al., 2018). Copyright @ 2018 American Chemical Society.

To investigate the cause of the high kinetic activation barrier of water oxidation, several possible reaction mechanisms were proposed, such as the oxide pathway, electrochemical oxide pathway, and hydrogen peroxide pathway.^47^ The widely accepted AEM is the one proposed by Nørskov group based on DFT calculations (Figure 2A).^35^^,^^48^^,^^49^^,^^50^ It involves four proton-electron transfer reactions centered on the metal active sites in acidic environment, which could be described as follows.^50^

(Equation 1) $$*+H_{2}O\;\to \text{HO}*+H^{+}+e^{–}\;\;\;\;\;\;\;\;\;\Delta G_{1}=\Delta G_{HO*}-\Delta G_{H_{2}O}-eU+k_{b}T\;\ln\;\alpha_{H^{+}}$$

(Equation 2) $$\text{HO}*\;\to \;O*+H^{+}+e^{–}\;\;\;\;\;\;\;\;\;\Delta G_{2}=\Delta G_{O*}-\Delta G_{HO*}-eU+k_{b}T\;\ln\;\alpha_{H^{+}}$$

(Equation 3) $$O*+H_{2}O\;\to \;\text{HOO}*+H^{+}+e^{–}\;\;\;\;\;\;\;\;\Delta G_{3}=\Delta G_{HOO*}-\Delta G_{O*}-eU+k_{b}T\;\ln\;\alpha_{H^{+}}$$

(Equation 4) $$\text{HOO}*\;\to \;O_{2}+*+H^{+}+e^{–}\;\;\;\;\;\;\;\;\;\Delta G_{4}=\Delta G_{O_{2}}-\Delta G_{HOO*}-eU+k_{b}T\;\ln\;\alpha_{H^{+}}$$

where ∗ represents the intermediate species adsorbed on the active sites. Clearly, each step requires Gibbs free energy. The step with the largest Δ*G* is termed as RDS, which governs the catalytic performance of OER process. For most OER reactions, RDS is mainly related to the HO∗ deprotonation step (Δ*G*_2_) or HOO∗ formation step (Δ*G*_3_), and the Δ*G*_O∗_ -Δ*G*_HO∗_ descriptor is used to predict catalytic activity.^50^ For an ideal catalyst, it is well known that the standard equilibrium potential for oxygen evolution is 1.23 eV, indicating the minimum value of Δ*G*_HOO∗_ -Δ*G*_HO∗_ is 2.46 eV. Whereas real catalysts are unable to meet this requirement and usually need an additional voltage to drive the reactions, that is, overpotential. Koper identified the universal scaling relation between HO∗ and HOO∗ with a constant of approximately 3.2 eV (Δ*G*_HOO∗_ -Δ*G*_HO∗_ = 3.2 eV ± 0.2 eV).^51^ From this, the minimum overpotential was calculated to be 0.37 eV by (3.2–2.46)/2. Moreover, the binding free energy of reaction intermediates and the Δ*G*_O∗_ -Δ*G*_HO∗_ descriptor clearly followed the “volcano plot” activity trend, which is a powerful and low-cost method to predict the OER performance of catalytic materials.^50^^,^^51^ While this scaling relationship may provide theoretical guidance for rapid screening of electrocatalysts (Figure 2B), it also hinders the design of catalysts due to the limited optimal catalytic materials are located at the top of the volcano plot. In a sense, therefore, breaking this scaling relationship is important for designing catalysts and reducing their overpotentials.^52^ Halck et al. demonstrated that such limitation can be eliminated through the modification of active sites.^53^ It was found that Ni-doped RuO_2_ displayed more potent activity than conventional RuO_2_, exceeding the predictions of the scaling relationship. This was attributed to the activation of *bridge* site as the proton donor-acceptor due to the introduction of Ni. Therefore, the donor-acceptor functionality should be effectively introduced into the oxygen evolution process as an additional descriptor, as it represented a simple multidimensional optimization of the multi electron catalytic process of water electrolysis. Based on the DFT calculations,^50^ the second charge transfer reaction was used as an additional descriptor to describe this donor-acceptor effect (Figure 2C).^53^

### Lattice oxygen evolution mechanism

Unlike traditional AEM where oxygen adsorption occurs on the interfacial metal active sites, LOM breaks the scaling limitation by involving the lattice oxygen in OER process (Figure 2D).^35^ Furthermore, the OER activity in numerous catalysts is pH-dependent and higher than that predicted by AEM, such as La_2_LiIrO_6_.^54^^,^^55^ The unconcerted proton-electron transfer steps of LOM can be concluded as follows

(Equation 5) $$*+H_{2}O\;\to \text{HO}*+H^{+}+e^{–}$$

(Equation 6) $$\text{HO}*\;\to \;O*+H^{+}+e^{–}$$

(Equation 7) $$O*+O_{L}\;\to \;O_{2}+V_{O}$$

(Equation 8) $$V_{O}+H_{2}O\;\to \;H*+H^{+}+e^{–}$$

(Equation 9) $$H*\;\to *+H^{+}+e^{–}$$

Clearly, the third step of LOM is significantly different from that of AEM. For LOM, the O=O bond is formed by coupling lattice oxygen (O_L_) to O∗ without the production of HOO∗, leading to O_2_ formation and an oxygen vacancy (V_O_) creation (Equation 7). Next, the resulting V_O_ is filled by H_2_O or the lattice oxygen migrating from the bulk to the surface to produce HO∗ species (Equation 8), revealing the origin of the surface reconstruction, amorphization, and dissolution of metal cations. Finally, after the deprotonation and regeneration of the metal active site (Equation 9), the reaction proceeds to the next cycle. Shao-Horn group experimentally demonstrated the presence of LOM in perovskite for the first time using *in situ* ^18^O isotope labeling mass spectrometry.^56^ And they pointed out that metal-oxygen covalency was closely related to OER activity and reaction mechanism. Typically, stronger covalency will lead to lower oxygen vacancy formation energy and faster oxygen exchange kinetics, thus activating lattice oxygen to participate in OER and triggering the proton-electron transfer of electrocatalytic process (Figure 2E).^56^^,^^57^^,^^58^ Therefore, LOM is especially prevalent in some heteroatom-doped Ru/Ir oxides, perovskites, and oxygen vacancy-rich electrocatalysts that are more prone to form oxygen vacancies.^57^^,^^58^^,^^59^ For instance, IrNi@IrO_x_ core-shell nanoparticles showed excellent catalytic activity, which was attributed to the metal vacancies induced by nickel leaching leading to a strong Ir-O covalency through LOM pathway.^60^ Although the LOM pathway involving lattice oxygen induces catalyst dissolution, the resulting metal vacancies also contribute to a significant decrease in the OER overpotential.^61^ In addition, it was found that some electrocatalysts followed by LOM pathway offered lower overpotential than the minimum theory value defined in AEM, as evidenced by the research on the theoretical performance of different perovskites.^44^ Unsurprisingly, compared to AEM, LOM volcano displayed a higher peak (Figure 2F).

### Oxide path mechanism

In a very different pathway, the oxide path mechanism (OPM) only involves O∗ and HO∗ intermediates, in which the peroxide generation step deprotonated by the hydroxide group is absent.^62^^,^^63^ The steps of OPM are as follows

(Equation 10) $$*+H_{2}O\;\to \text{HO}*+H^{+}+e^{–}$$

(Equation 11) $$2\text{HO}*\;\to \;O*+*+H_{2}O$$

(Equation 12) $$2O*\;\to \;O_{2}+2*$$

**Figure 3:**
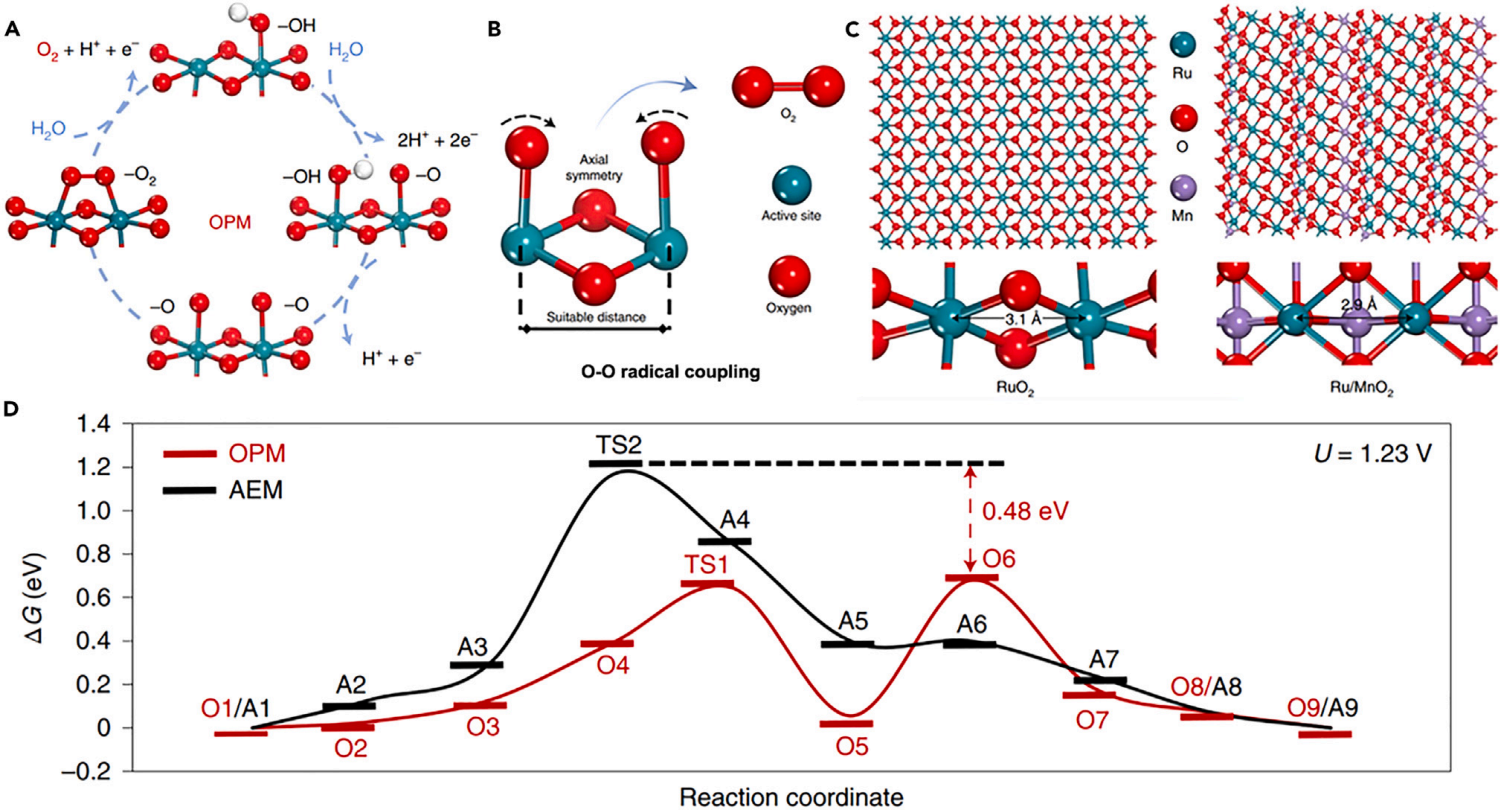
Schematics of OPM mechanism for Ru-based OER electrocatalyst Reproduced with permission from (Lin et al., 2021). Copyright @ 2021 Nature Publishing Group. (A) Schematic illustration of simplified OPM mechanism. (B) O–O radical coupling promoted by symmetric dual active sites. (C) Crystalline structure models of RuO_2_ and α-MnO_2_ with Ru atoms substituting for the Mn at surface sites. (D) The free energy (ΔG) diagrams of AEM and OPM at 1.23 V versus RHE. States O1–O9 and A1–A9 present the different elementary states in the OPM and AEM pathways, respectively.

Obviously, during the OPM process, O-O radical is directly coupled to produce O_2_ (Figure 3A), which does not require the involvement of lattice oxygen compared to LOM, avoiding the generation of oxygen vacancy defects, and thus addressing the metal dissolution issue of LOM-based electrocatalysts to some extent.^64^ In contrast to AEM, this OPM process breaks the scaling relationship of reaction intermediates arising from the absence of additional absorbates (HOO∗) formation, shortening the reaction pathway. However, the OER process under the OPM pathway can dissociate water and trigger the O-O radical coupling to produce O_2_ only when adjacent metal ions with appropriate atomic distances act synergistically. Therefore, OPM has stricter requirements for the geometrical configuration of metal active sites compared to AEM and LOM.^65^^,^^66^ Symmetric bimetallic sites close enough are prone to initiate O-O radical coupling at a low energy barrier (Figure 3B).^64^ For example, Lin et al. prepared a crystalline α-MnO_2_ nanofiber-supported Ru electrocatalyst (Ru/MnO_2_) by one-step cation exchange method, in which Ru atoms followed the periodic arrangement of Mn sites in α-MnO_2_ crystals.^64^ Aberration-corrected high-angle annular dark-field scanning transmission electron microscopy (HAADF-STEM) analysis revealed that the interatomic Ru-Ru distance (2.9 Å) in Ru/MnO_2_ matched the theoretical interatomic distance of 2.9 Å for Mn-Mn, which was shorter than that in RuO_2_ (3.1 Å) (Figure 3C), facilitating O-O radical coupling. Such OPM-dominated OER reaction process enables the electrocatalyst to overcome the overpotential limitations imposed by the conventional AEM pathway (Figure 3D). Ultimately, Ru/MnO_2_ harvested an intrinsic OER activity more than 600 times higher than that of RuO_2_.

## Degradation mechanisms

Due to the strong corrosive environment and applied high voltage, OER electrocatalysts will inevitably undergo drastic surface structural or compositional changes, and may even cause accelerated catalyst degradation. On the other hand, the fluid flow in the electrolyzer can accelerate the interfacial mass transfer and diffusion between the catalytic materials and corrosive media, leading to an increase in the dissolution rate of some electrocatalysts. As a result, this mechanical impact can also exacerbate the decay of catalytic materials, thus reducing their durability. Overall, the stability of anode catalysts remains an important factor hindering the practical application of PEM. In this sense, the exploration of the degradation mechanisms of catalytical materials is important for the design of electrocatalysts with strong stability.

### Electrocatalysts decay

**Figure 4:**
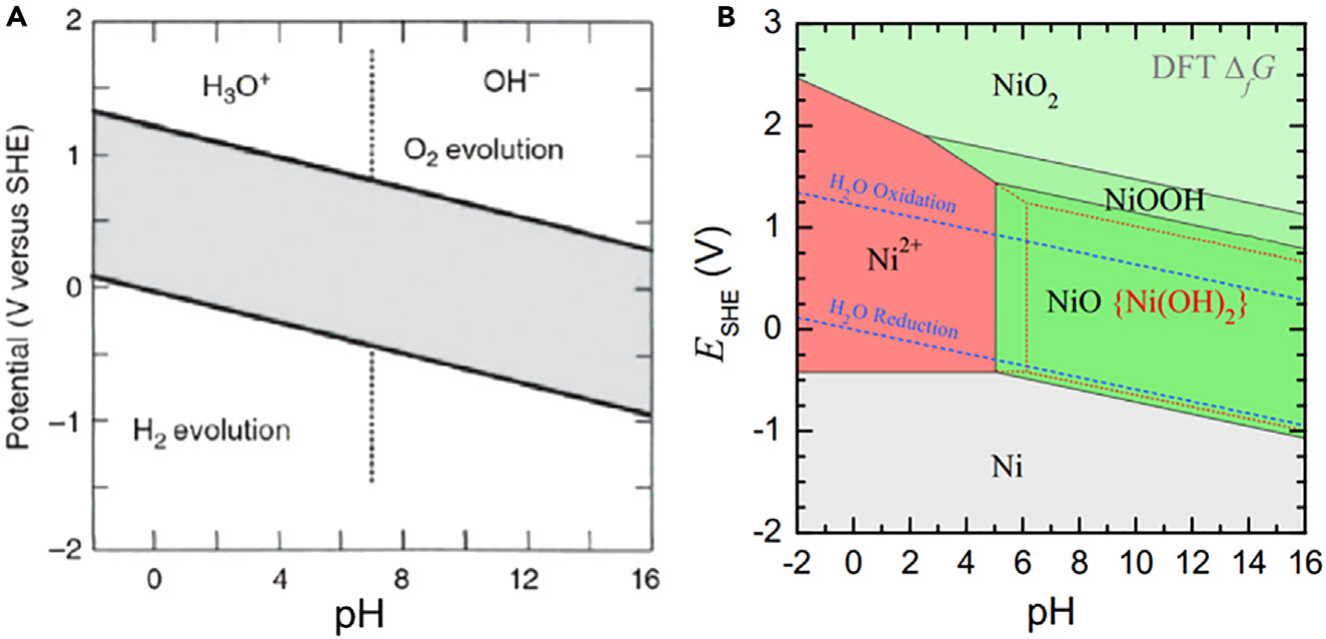
Thermodynamic stability evaluation of materials by Thermodynamic stability evaluation of materials under OER conditions (A) Pourbaix diagram for water. The gray area represents the thermal dynamically stable region of water. Reproduced with permission from (Eliaz et al., 2019). Copyright @ 2019 Wiley-VCH Verlag GmbH & Co. KGaA, Weinheim. (B) Pourbaix diagram of the Ni electrode. Reproduced with permission from (Huang et al., 2017). Copyright @ 2017 American Chemical Society.

Owing to the harsh acidic OER catalytic environment, some metals are easily induced to dissolve. According to the Pourbaix diagram, the electrochemical stability of the catalytic materials in acidic electrolyte can be described by comparing the standard Gibbs free energy of the related species under the specific voltage and pH.^67^ More specifically, the dynamic process of electrocatalysts under catalytic conditions may be deduced from the Pourbaix diagram. In general, metal-based materials should be stable in a narrow domain of moderate pH and low potentials (Figures 4A and 4B).^68^^,^^69^ When exposed to the acidic solutions, metals can be spontaneously oxidized or even dissolved into metal ions as an increase in applied potential, while the phase transition is further induced and OER proceeds. Consequently, metal-based electrocatalysts decay is driven by the corrosive chemical environment and high anodic voltage.

The morphology and composition of catalytic materials are the critical intrinsic instability factors. Gibbs-Thomson equation disclosed that the dissolution potential and cohesion energy of nanoparticles decrease with decreasing particle size, thus inducing metal dissolution.^70^ Benefiting from the favorable surface modification caused by intense Ni leaching, Ir-Ni oxide thin film harvested 20 times higher mass activity than IrO_2_ during the initial OER process.^71^ In this active oxide film, they pointed out that the surface catalytic activity would enhance significantly when the residual Ni ratio was essentially constant at 12 at% Ni. These residual Ni may be stabilized in the oxide matrix through the interaction with Ir. As a result of electrochemical oxidation and continuous leaching of nickel atoms during the catalytic process, the morphology of the catalyst also underwent a dramatic change from nanoparticles to the core-shell structures possessing the iron-rich oxidized shell and metallic IrNi core.^72^ Coupled with X-ray photoelectron spectroscopy (XPS) and X-ray absorption near edge structures (XANES) data, a surface structure similar to that of the thin film catalyst arising from the retention of Ni species in the surface area was found. Chronopotentiometric measurements regarding stability evaluation showed that the potential remained constant throughout the testing process, except for a slight increase in the first 30 min of the test, indicating the stable behavior of IrNi_3.3_ nanoparticles.^72^ During the acidic OER processes, Ru-based catalysts are prone to overoxidation to form soluble high-valent ruthenium ions (higher than 4-valent, e.g., RuO_4_), leading to poor catalytic stability. To address this issue, Zhang group tailored the electronic structure of RuO_2_ by the introduction of W and Er, largely improving the oxygen vacancy formation energy, and thereby inhibiting the dissolution of Ru species.^73^ Remarkably, the prepared W_0.2_Er_0.1_Ru_0.7_O_2-δ_ exhibited excellent stability with steadily operating for 120 h at 100 mA cm^−2^ current density in PEM device and 500 h at 10 mA cm^−2^ in acidic media. It is not unique, Pt-doped RuO_2_^74^ and PtCo-RuO_2_^75^ also employed similar strategy to prohibit the dissolution and overoxidation of Ru species by tuning the electronic structure of RuO_2_ through charge redistribution during OER cycles.

### Support degradation

To maximize catalytic materials utilization, nanomaterials in electrochemical devices, especially powdered ones, are generally anchored to a conductive support to form uniformly dispersed electrodes. In this sense, the physical properties of the support and its interaction with the active material play an important role in the long-term stability of OER catalysts. For example, conventional carbon-based support materials are prone to passivation and particle detachment induced from the weak support-catalyst interaction in corrosive acidic and oxidizing environment. Choi et al. demonstrated the major degradation pathway for the carbon-based substrate in the FeN_x_C_y_ catalyst, carbon oxidation would occur under the applied anodic potential higher than 0.9 V with the destruction of active sites.^76^ However, the degradation rate of the defective RuO_2_ catalyst supported on carbon cloth (CC) was much lower than that on the glassy carbon electrode during the 20 h of CP test. It had been proved that the strong catalyst-support interaction contributed to this superior stability of RuO_2_ supported on CC.^77^ Particularly, *in situ* generated substrates were more favorable for anchoring active species to improve catalyst stability. The IrO_x_/SrIrO_3_ catalysts prepared by the Jaramillo group showed excellent OER performance with an overpotential of only 270 to 290 mV at 10 mA cm^−2^ during 30 h of continuous test in acidic medium.^78^ This was attributed to the formation of highly active surface layers of IrO_3_ or anatase-type IrO_2_ species through the strontium leaching from the surface layer of the substrate SrIrO_3_ film.^78^

To a certain extent, strong interactions between the surface (groups) of the building carriers and the catalyst can inhibit particle detachment or dissolution during electrocatalysis. Metal oxides have been considered as the alternative stable supports. Especially for metal electrocatalysts, it has been found that the strong oxide-metal interaction was favorable to the stability improvement of catalytic materials. For example, based on the high corrosion and electrochemical oxidation resistance of antimony doped tin oxide (ATO), Liu et al. prepared iridium oxide catalysts employing ATO nanowires synthesized by electrospinning as the support.^79^ Owing to the porous structure and high conductivity of ATO, an ultralong continuous acidic OER process at a current density of 450 mA cm^−2^ for over 640 h can be offered by the supported IrO_2_. More recently, Shan et al. synthesized Ir_0.06_Co_2.94_O_4_ catalyst by anchoring iridium single atoms into the cationic sites of cobalt spinel oxide, which maintained continuous OER electrocatalysis for more than 200 h under high corrosive and oxidative environments.^80^ Such superior stability was attributed to the strong interaction between iridium and cobalt oxide support stemming from the modified electronic structure of catalyst in acidic electrolyte, which was also the fundamental reason for the improvement of corrosion resistance.

### Catalyst detachment

In general, ionomer binders are employed to uniformly distribute and fix the powdered catalytic materials on the electrodes. During the electrolysis process, the binder may be degraded by nucleophilic attack from water or free radical oxygen oxidation, resulting in a decrease in mechanical toughness of membrane electrode and thereby poor stability.^81^ Additionally, too high mass loading is prone to weaken the bind of catalytic materials with electrode, leading to the detachment of catalyst.^82^

As everyone knows, the water splitting process is always accompanied by the production of gas, such as H_2_ and O_2_. However, owing to the limited solubility in the electrolyte, these bubbles may block catalytic active sites and decrease the electrolysis efficiency. Especially for porous nanomaterials, bubbles will accomplish nucleation, growth, and separation at heterogeneous interfaces such as pores, which may not only cover the active sites, block ionic conduction pathway, but also worsen the mechanical stability of electrocatalysts.^83^ Moreover, during the stripping process of bubbles, the heterogeneous nucleation sites previously occupied by bubbles will be inactivated as the pores of catalytic materials are gradually filled with electrolyte. As the current density increased, it was observed that the generation of bubbles became stronger, which may damage the interface between the catalytic materials and the electrode.^84^ In contrast to low current densities, only a small amount of metal sites dissolved at high current densities (more than 1000 mA cm^−2^), while most active metal species underwent physically detachment as solid precipitates. In addition, the fluid flow inside the electrolyzer can flush the electrode and cause the catalytic materials to fall off, accelerating the decay of catalysts therefore reducing their durability.

## Rational design of electrocatalysts through electronic structure regulation

### Alloy engineering

Adjusting the catalytic performance of electrocatalysts by alloying other elements is generally considered an effective strategy, which can optimize the binding strength of oxygen intermediates through electronic synergistic effects.^71^^,^^85^^,^^86^^,^^87^ Drawing from Guo team’s research, alloying of Ir with 3D transition metals, such as Ni and Co, can drive the distribution of d-band electrons away from the Fermi energy level, making adsorption energy of oxygen intermediates weaker, and eventually yielding much-enhanced OER activity.^88^ The optimized IrCoNi porous hollow nanocrystal delivered a remarkable catalytic activity with the overpotential of 303 mV at 10 mA cm^−2^ in acidic media, 10 times higher than that of Ir/C catalyst.^88^ Similarly, Ir_0.60_Cu_0.40_ alloy microspheres prepared by Deng group provided a high electrocatalytic activity with a low overpotential of 255 mV at 10 mA cm^−2^ in 0.1 M HClO_4_.^86^ Combining the analysis results of XPS and X-ray diffraction (XRD), alloying of iridium with metallic copper optimized the d-orbital distribution of the 5d electrons of iridium, thus modifying the electronic structure of iridium, which in turn improved the OER performance of catalytic material. Downsizing the nanoparticles (NPs) of catalysts can increase the number of active sites while improving the utilization of active atoms. Guo et al. prepared a series of sub-10 nm Rh−Ir alloy NPs with tunable compositions employing a microwave-assisted technique for efficient and stable water oxidation in acidic media.^89^ The transmission electron microscopy (TEM) analysis results revealed that the particle size of Rh-Ir alloy NPs increased linearly with Rh content, indicating a direct correlation with the composition of catalyst. When coupled with DFT calculations performed on the more stable (111) surface of Rh, Rh_0.25_Ir_0.75_, and Ir NPs, a weaker O binding of Ir_3_ site on Rh_0.25_Ir_0.75_ resulting from the deviation from the scaling relationship between O and OH binding energies was demonstrated. This not only reduced the binding energy difference between the O∗ and HOO∗ intermediates, but also accelerated the kinetics of RDS. Obviously, alloying small amounts of Rh into Ir led to a substantial enhancement of OER activity under acidic conditions, as well as the excellent stability with a minor overpotential increase (13 mV) after 2000 cyclic voltammetry (CV) cycles.^89^

**Figure 5:**
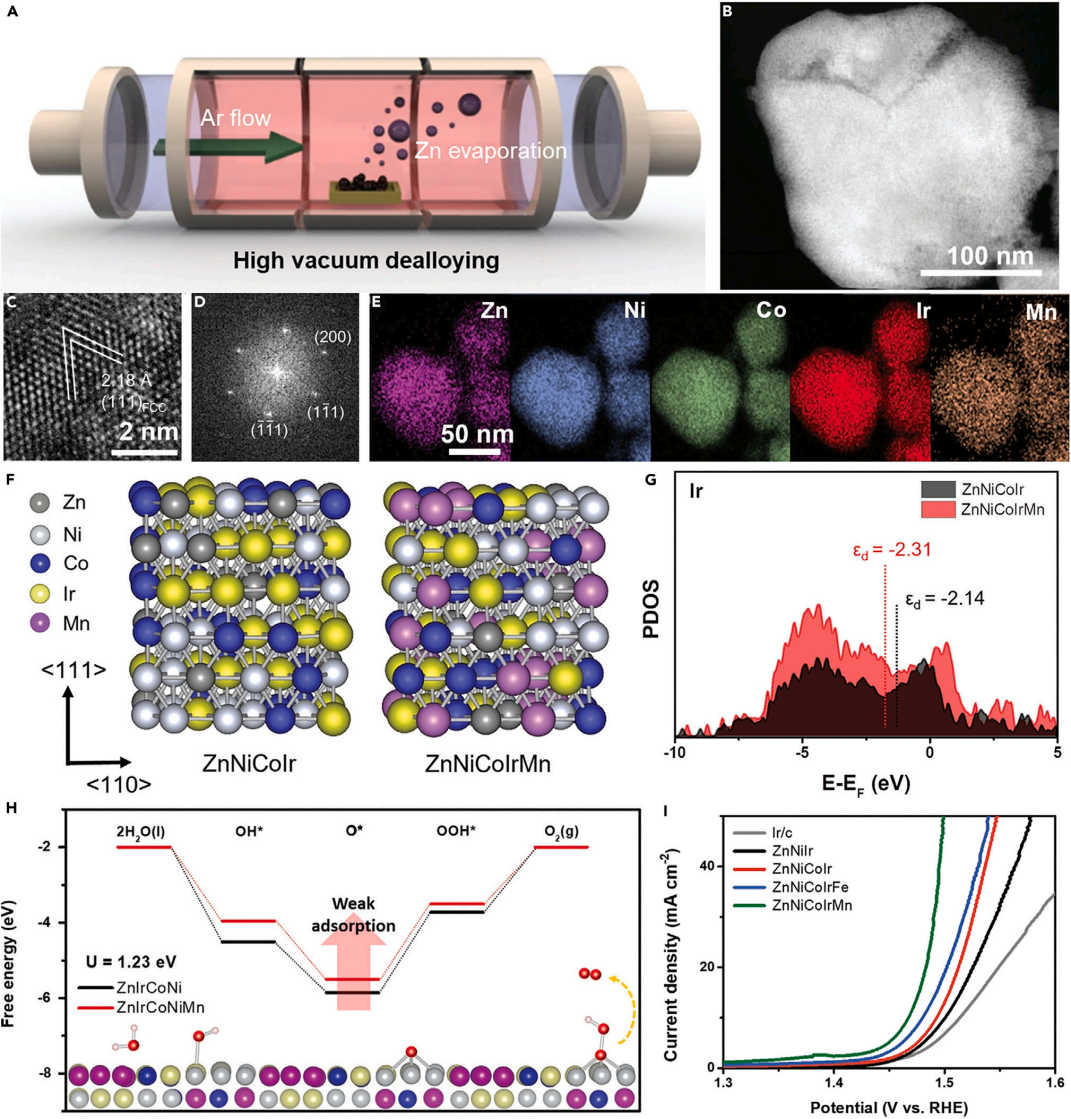
The designing strategy and characterization of the prepared ZnNiCoIrMn Reproduced with permission from (Kwon et al., 2023). Copyright @ 2023 Wiley-VCH GmbH. (A) Scheme of dealloying process of ZnNiCoIrMn. (B) STEM image of ZnNiCoIrMn. (C) HRTEM image of ZnNiCoIrMn. (D) Selective area electron diffraction (SAED) pattern of ZnNiCoIrMn. (E) The energy dispersive X-ray (EDX) elemental mapping of ZnNiCoIrMn. (F) Computational modeling of ZnNiCoIr and ZnNiCoIrMn with random atomic arrangement. (G) d-band projected DOS (pDOS) of Ir for ZnNiCoIr and ZnNiCoIrMn. (H) Free energy diagram of ZnNiCoIrMn. (I) LSV curves of OER on various materials in 0.5 M H_2_SO_4_ with scan rate of 1,600 rpm.

Combining five or more elements in relatively high concentrations to produce new materials, known as high-entropy alloys (HEAs).^90^ Multiple studies have shown that HEAs possess superior properties beyond those of conventional alloys, such as excellent corrosion resistance, exceptional ductility, and high electronic structure tunability.^90^^,^^91^^,^^92^^,^^93^^,^^94^ The electron density redistribution around Ir was achieved in the preparation of ZnNiCoIrMn HEAs with high crystallized nanoporous structure by sacrificing zinc element, which tailored the electronic structure of active Ir sites (Figures 5A–5F).^95^ With the doping of Mn, ZnNiCoIrMn generated a wider d-band structure than ZnNiCoIr, resulting in the d-band center being far away from the Fermi energy level, which effectively reduced the energy barrier of RDS (i.e., HOO∗ formation), and consequently improved OER activity (Figures 5G–5I). As evidenced by XPS and composition analysis, the elements with weaker adsorption energy in the alloy were difficult to be solvated, and only the transition metals on the surface were tend to dissolve, leading to the formation of ir-rich surface, which explained the enhanced durability of the OER under acidic conditions. In addition, the lattice distortion caused by the different lattice parameters of elements could produce a slow diffusion effect, preventing the dissolution of elements and enhancing the stability of catalyst.^95^ HEAs also suffer from dissolution under acidic OER conditions. IrFeCoNiCu HEA NPs on carbon paper synthesized by microwave-assisted shock approach exhibited high acidic OER activity.^96^ As revealed by TEM, during the electrochemical process, an active Ir rich shell characterized by nano-domains rapidly formed on IrFeCoNiCu HEA NPs surface, which was mainly attributed to the dissolution of 3D metals in the alloy. Fortunately, the core of NPs still maintained a homogeneous single-phase HEA structure.

According to these reported literatures, strong synergistic electronic interactions formed by alloying Ir or Rh with transition metals on the nanoscale can weaken the binding energy of the surface intermediates by shifting the d-band away from the Fermi energy level, leading to enhanced OER activity. Unfortunately, metal dissolution and surface reconstruction that arises from the applied high voltage in highly corrosive electrolyte may deteriorate the performance and durability of OER process. To achieve a balance between catalytic activity and stability, it is necessary to adopt strategies such as the construction of strong coordination bonds and morphology control to adapt the catalyst to the transformation of the electrochemical environment through changes in the atomic and electronic structures. This can make this poisoning effect controllable and enable the OER process to reach dynamic stability as soon as possible.

### Heteroatom doping

**Figure 6:**
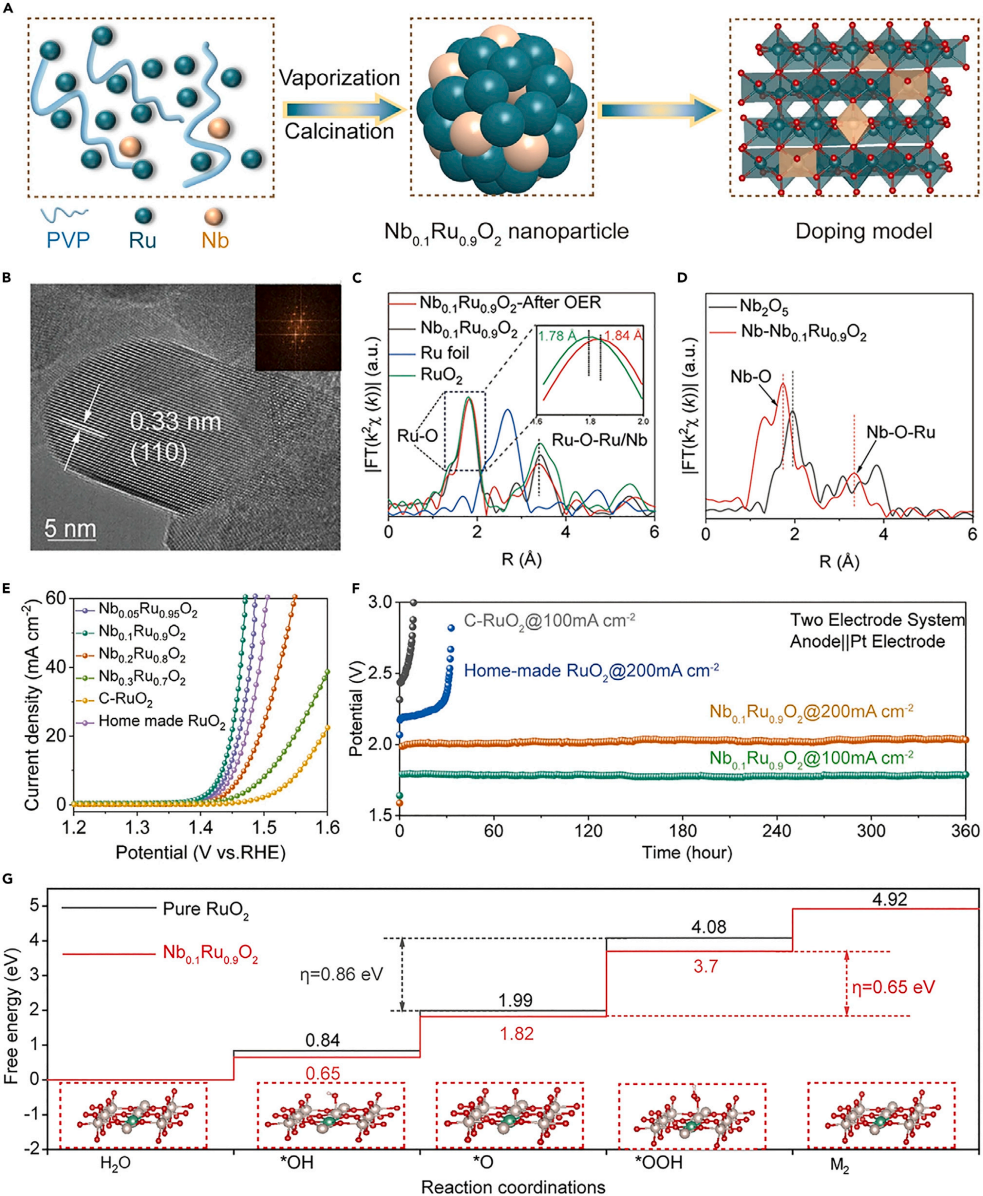
The designing strategy and characterization of the prepared Nb-RuO_2_/G Reproduced with permission from (Liu et al., 2023). Copyright @ 2023 Elsevier Inc. (A) Scheme of synthetic route of Nb_0.1_Ru_0.9_O_2_. (B) HRTEM and FFT (inset) images of Nb_0.1_Ru_0.9_O_2_. (C) Fourier transformed Ru K-edge EXAFS in R spaces of various materials. (D) Fourier transformed Nb K-edge EXAFS of Nb_0.1_Ru_0.9_O_2_ and Nb_2_O_5_ in R spaces. (E) LSV curves of OER on various materials in 0.5 M H_2_SO_4_ with scan rate of 1,600 rpm. (F) Chronopotentiometry tests of various materials at 100 and 200 mA cm^−2^. (G) Free energy diagram of Nb_0.1_Ru_0.9_O_2_ and RuO_2_.

Heteroatom doping is a versatile pathway to tune the electronic structure of catalysts.^97^^,^^98^^,^^99^ For example, Shan et al. synthesized Co-doped RuIr alloy with boost OER performance using a co-reduction polyol method.^99^ This remarkable catalytic property of catalyst is attribute to the increased concentration of O^I−^ species and weakened adsorption of oxygen intermediates resulting from the inevitable Co leaching from Co-doped RuIr alloy.^99^ In order to avoid the dissolution of low valence metals and over-oxidation of Ru at high current density (>100 mA cm^−2^),^100^ Liu et al. introduced the high-valent transition metal Nb into RuO_2_ to prepare Nb–RuO_2_ NPs toward active and stable acidic OER (Figure 6A).^101^ TEM, fast Fourier transformation (FFT) and selected area electron diffraction (SAED) investigations revealed that Nb-doped RuO_2_ NPs possessed a broader lattice distance compared to RuO_2_, together with the atomic dispersion of Nb in RuO_2_ (Figure 6B). As demonstrated by DFT calculations, owing to the electrons transfer from Nb^4+^ to Ru via bridging oxygen after Nb doping, more low-valent Ru sites were generated, which led to an increase in the electron density around Ru sites. Meanwhile, the coordination environment of Nb K-edge in representative Nb_0.1_Ru_0.9_O_2_ also confirmed the increased electron density around Ru sites, which originated from the weakened Ru-O covalency by strong Nb-O band (Figures 6C and 6D). This electron modulation facilitated the protection of Ru sites from over-oxidation and optimized the adsorption of reaction intermediates, thereby improving the OER catalytic performance (Figures 6E–6G).^101^

Non-metallic elements with greater electronegativity tend to form stronger covalency with metals, which facilitates the charge transfer. Thus, anion doping may modulate the surface property and electronic structure of electrocatalysts, thereby optimizing the adsorption energy of the intermediates and boosting the OER process.^102^^,^^103^ For example, S-doped monoclinic SrIrO_3_ (M-SrIrO_3_) nanosheets synthesized by sol-gel and sulphuration methods exhibited superior OER activity and durability in strong acidic media.^104^ TEM and energy dispersive spectroscopy (EDS) elemental mapping analysis confirmed that Sr cations leached from the catalyst surface during the actual OER process, leading to the surface reorganization of amorphous Ir(O, S)_x_. This may be an important reason for the sharp decrease of lattice O with the leaching of Sr. These results indicated that the surface-reorganized amorphous Ir(O, S)_x_ had abundant oxygen defects, which was favorable for OER kinetics. When coupled with DFT calculations, the conclusion that S doping promoted the electronic conductivity of M-SrIrO_3_ was evidenced by the fact that S-doped M-SrIrO_3_ possessed much narrower band gap than that of undoped M-SrIrO_3_. Moreover, the introduction of S lowered the binding energy of intermediates on M-SrIrO_3_ surface, thereby promoting OER.^104^ As another example, by integrating boron species into RuO_2_ using a boric acid-assisted strategy, B-doped RuO_2_ (B-RuO_2_) structure with excellent acidic OER performance was prepared.^105^ Further theoretical calculations suggested that the formation of B-O covalency and exposure of the fully coordinately bridge ruthenium site (Ru-bri site) resulting from B doping could transform the inactive Ru-bri sites into OER-active sites and modulate the electronic structure of catalyst. This led to more exposed active sites and decreased energy barriers of intermediates, resulting in enhanced OER activity.^105^

Apparently, both cation and anion doping can contribute to enhanced overall OER performance. Specifically, the ligand and strain effects of the doped metals can modulate the local electronic density and optimize the coordination environment, decreasing the energy barriers of intermediates, thus improving the electrical conductivity and therefore faster OER kinetics. Anion doping is prone to induce the formation of covalent bonds, which modulates the electronic structure of catalysts, enabling faster electron transfer and leading to the optimization of reaction intermediates binding strength.

### Defect engineering

**Figure 7:**
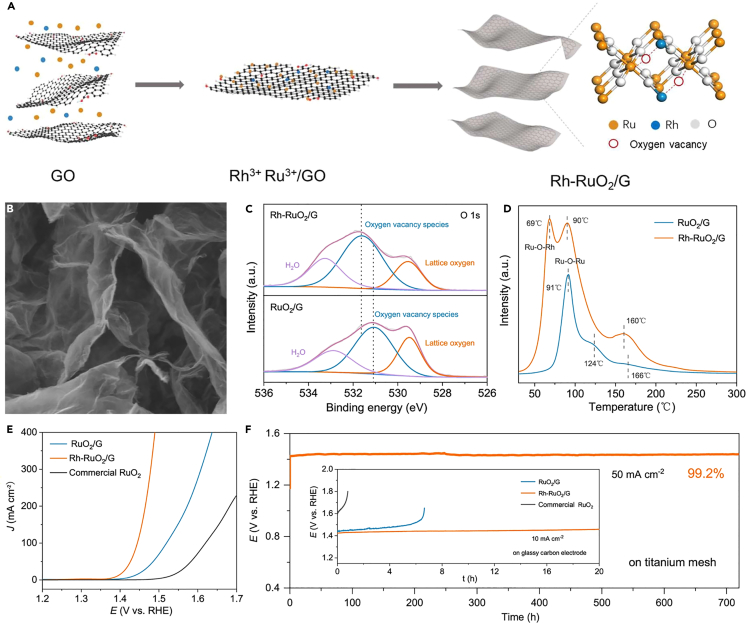
The designing strategy and characterization of Rh-RuO_2_/G Reproduced with permission from (Wang et al., 2023). Copyright @ 2023 Springer Nature Limited. (A) Scheme of synthetic route of Rh-RuO_2_/G. (B) SEM image of Rh-RuO_2_/G. (C) O 1s XPS spectra of Rh-RuO_2_/G and RuO_2_/G. (D) H_2_-TPR spectra of Rh-RuO_2_/G and RuO_2_/G. (E) LSV polarization curves of Rh- RuO_2_/G, RuO_2_/G, and commercial RuO_2_ in 0.5 M H_2_SO_4_. (F) The galvanostatic curves at a current density of 10 mA cm^−2^ and 50 mA cm^−2^.

Introduction of structural defects into catalytic materials is a common electronic modulation strategy to boost efficient water oxidation. Construction of grain boundaries, vacancies and adatoms are often employed to introduce the structural defects into catalytic materials to improve their electrocatalytic activity. It has been shown that the anionic defects such as oxygen vacancies can improve the electronic conductivity of catalysts by altering the coordination environment of metal active centers.^57^^,^^106^^,^^107^^,^^108^^,^^109^ For example, by leaching nickel, many lattice vacancies were injected into the iridium oxide shell, leading to the formation of defective IrNi@IrO_x_ core-shell NPs.^60^ Introduction of lattice vacancies resulted in a significant increase of d-band holes in the iridium center followed by oxygen vacancies doping. Therefore, owing to the lowered 5d energy of iridium, the electrophilicity of the surface oxygen ligands was enhanced, reducing the kinetic barriers during the OER catalytic process. This was conducive to the formation of O-O bonds through nucleophilic attacks on electrophilic oxygen by water molecules or hydroxyl ligands. Consequently, electrophilic oxygen as the catalytic active sites can promote a drastic increase in OER activity.^60^ Recently, to achieve a balance between activity and stability and avoid excessive oxidation of Ru-based materials, Wang et al. fabricated Rh-doped RuO_2_ on graphene (Rh–RuO_2_/G) by exchange adsorption method with the remarkable OER activity and stability (Figures 7A, 7B, 7E, and 7F). After exchanging the low oxidation state of Rh, abundant stable oxygen vacancies were generated on the surface of Rh–RuO_2_/G nanosheets to maintain charge neutrality (Figure 7C). This not only provided an effective charge compensation for the doped low valent cations, but also endowed the catalyst with enhanced electron trapping and transfer ability.^108^ ^18^O isotope labeling measurements evidenced that the lattice oxygen was absent from the oxygen production, suggesting that the conventional LOM did not dominate the OER process. As revealed by H_2_ temperature-programmed reduction (H_2_-TPR) analysis (Figure 7D) and DFT calculations, the formation of O∗ under the synergistic interaction of low valent Ru-O-Rh active sites and enriched oxygen vacancies was the RDS of Rh-RuO_2_/G, which followed the oxygen vacancy sites mechanism and broke the traditional reaction barriers (HOO∗) of RuO_2_ governed by AEM.^108^

In contrast, cationic defects may change the local electron distribution by triggering lattice distortion, thus optimizing the adsorption behavior of intermediates.^35^^,^^110^^,^^111^ Benefiting from the abundant Ru vacancies on the surface of RuO_2_ nanosheets, the ultra-thin RuO_2_ nanosheets prepared by molten salt method exhibited significant superior activity toward acidic OER.^111^ This was attributed to the weakened adsorption ability of O∗ and decreased energy conversion from O∗ to HOO∗. In another system, Zn-doped RuO_2_ fabricated by *in situ* electrochemical etching (E–Zn–RuO_2_) displayed superior OER activity and durability than that obtained by pure acid etching (C–Zn–RuO_2_).^112^ *In situ* electrochemical Zn leaching led to more defects, and such irreversible surface reconstruction was beneficial for exposing more active sites, enabling the catalyst to activate the pre oxidation of low valent Ru sites at relatively low working potential. Moreover, the doping of Zn resulted in the accumulation of electrons at the Ru sites, reducing the oxidation state of Ru. This not only weakened the adsorption of O on the Ru atoms, which was conducive to lower the energy barrier of the OER process, but also made the d-band central energy level of RuO_2_ closer to the Fermi level, shortening the Ru-O bond. The optimized electronic structure played a crucial role in the electrocatalytic activity of catalyst. The shortened Ru-O bond was the main reason for the extended lifetime of E–Zn–RuO_2_.^112^

Obviously, defect engineering can optimize the adsorption behavior of intermediates by inducing lattice distortion or changing the coordination environment of active metal centers to alter local electron distribution. It should be noted that anionic defect engineering, especially the introduction of oxygen vacancies, can promote lattice oxygen migration, thereby accelerating surface amorphization during the OER process.^108^ The formation of the amorphous surface indicates the lattice instability of the catalyst under OER environments, and this LOM pathway involving lattice oxygen is often considered an important reason for metal dissolution. However, it has been shown that the surface reconstruction process is governed by the coordination environment,^113^ which can terminate this structural transformation to a stable oxide layer rather than endless leaching. That is, there are strong correlations between surface structure reconstruction and electronic structure of electrocatalysts. The identification of these correlations will guide the researchers to design more promising catalysts.

### Surface reconstruction

**Figure 8:**
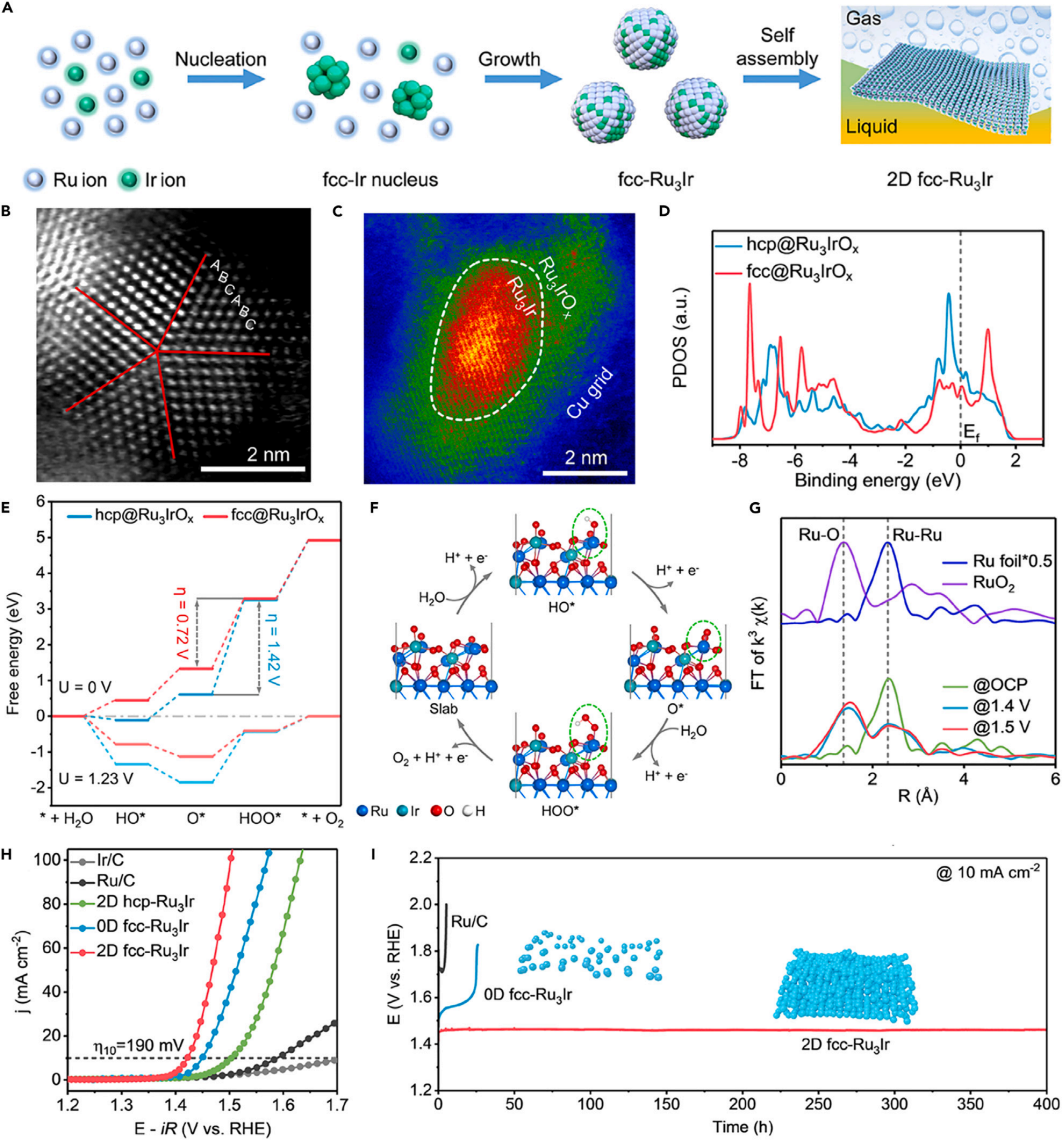
The illustration and characterization of 2D fcc-Ru_3_Ir Reproduced with permission from (Fan et al., 2023). Copyright @ 2023 American Chemical Society. (A) Scheme of the synthesis process of 2D fcc-Ru_3_Ir. (B) Atomic-resolution HAADF-STEM image of 2D fcc-Ru_3_Ir. (C) False-colored HAADF-STEM image of 2D fcc-Ru_3_Ir after OER. (D) PDOS of Ru 3d orbitals in the fcc@Ru_3_IrO_x_ and hcp@Ru_3_IrO_x_. (E) OER free energy diagrams of fcc@Ru_3_IrO_x_ and the hcp@Ru_3_IrO_x_ under the applied potentials, respectively. (F) Atomic structures of the OER intermediates on the fcc@Ru_3_IrO_x_. (G) k^3^-weighted FT-EXAFS of the Ru K-edge for 2D fcc-Ru_3_Ir under different applied potentials of OCP. (H) OER polarization curves of various materials in Ar-saturated 0.5 M H_2_SO_4_ electrolyte. (I) Chronopotentiometry tests and schematic models (insets) of various materials.

More and more research has manifested that many electrocatalysts may undergo surface reconstruction during the catalytic process, especially in harsh acidic corrosion environments for OER, yet it can be advantageously used to improve electrocatalytic performance.^89^^,^^114^^,^^115^ Owing to this controlled surface reconstruction, the pristine material needs to undergo spontaneous atomic and electronic structure changes to adapt to the electrochemical environment shift, and the new species generated during the electrocatalytic process serves as the real active sites.^116^ For example, the leaching of strontium from surface layer of SrIrO_3_ thin films resulted in the formation of IrO_x_/SrIrO_3_, and the resulting highly active surface layers contributed to the excellent OER performance in acidic electrolyte.^78^ Obviously, the surface reconstruction can be triggered by some cation leaching under the driving of corrosive chemical environment and high anodic potential. It has been shown that lattice instability has an important impact on the surface reconstruction of catalysts, and materials with lower coordination number or bond strength are more prone to transformation.^117^ Based on the synthesis of RuMn alloy by doping Mn atoms on carbon fiber paper, An and co-workers effectively improved the durability and activity of the catalytic materials through further surface reconstruction.^117^ It was observed that RuMn alloy generated an amorphous RuO_x_ surface after OER electrochemical test. Compared to RuCr, RuCo, RuZn alloys, Mn in RuMn alloy had a stronger effect on the electronic structure of Ru, as was evidenced by XPS analysis. This indicated that the Mn doping resulted in higher bond strength of Ru, which facilitated RuMn alloy to undergo moderate dissolution and favored further oxidation and the insertion of oxygen atoms, leading to a highly disordered network of surface RuO_x_. Such stable amorphous surface not only can protect the internal alloy, but also inhibited steady-state dissolution, ensuring the high OER durability of catalyst for 720 h under acidic condition.^118^ This study also validates the claim that the level of surface reconstruction is controllable and will terminate in a stable oxide layer rather than endless leaching.^119^ Cui and Zheng group investigated the OER performance of quasi-two-dimensional Ru_3_Ir nanocrystals with an unconventional face-centered cubic (fcc) phase (2D fcc-Ru_3_Ir) synthesized by self-assembled engineering and phase modulation (Figure 8A).^120^ The false-colored HAADF-STEM and XPS results demonstrated that the surface atoms of 2D fcc-Ru_3_Ir were reconstructed to active amorphous Ru_3_IrO_x_ during the acidic OER process (Figures 8B and 8C). Benefiting from the electronic structure modulation of Ru_3_IrO_x_ by fcc-Ru_3_Ir substrate, the density of Ru 3d states near Fermi level of fcc-Ru_3_Ir@Ru_3_IrO_x_ was reduced, lowering the energy barrier of O∗→HOO∗ RDS, and thus boosting the OER activity (Figures 8D–8F and H). Furthermore, when coupled with XANES analysis, it was found that such 2D self-assembled structure was able to suppress the excessive dissolution of surface Ru_3_IrO_x_ during the OER process (Figure 8G), and thereby improving the corrosion resistance durability of catalyst (Figure 8I).^120^

Overall, controlled surface reconstruction driven by electrocatalytic conditions can change the electronic structure of pristine materials and generate new active species. Specifically, the modulation of the electronic density of states near the Fermi level of the active centers lowers the reaction energy barrier of RDS, boosting the OER performance. Most notably, to maintain the high performance of catalysts, necessary structural modification strategies should be taken to prevent the catalytic materials from transition oxidation to soluble metal ion derivatives governed by LOM pathway.

### Metal-support interaction effect

**Figure 9:**
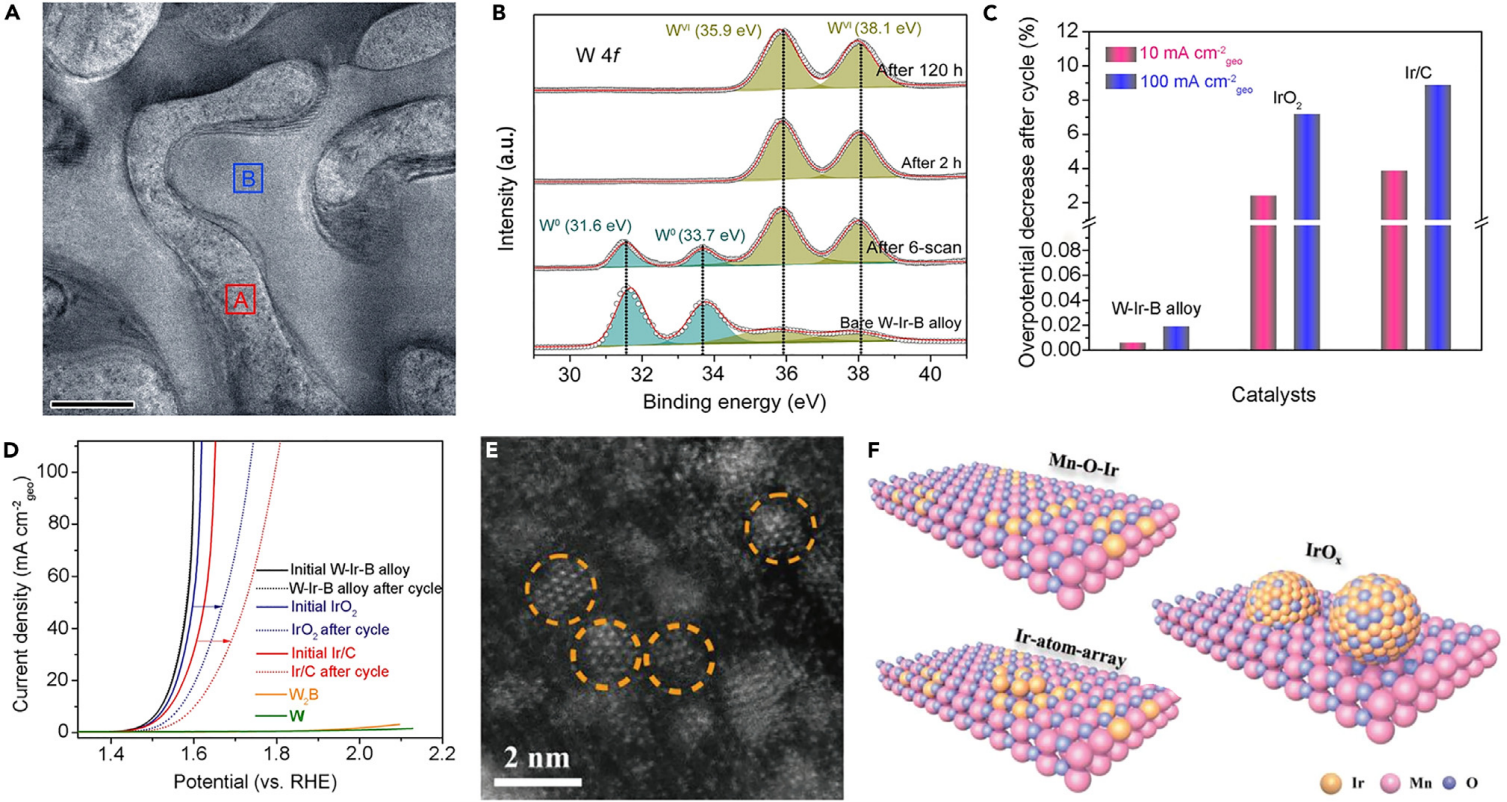
The characterization of W-Ir-B alloy (Reproduced with permission from (Li et al., 2021) Copyright @ 2021 Springer Nature Limited) and Ir-MnO_2_(160)-CC (Reproduced with permission from (Weng et al., 2023). Copyright @ 2023 Advanced Science published by Wiley-VCH GmbH). (A) TEM image of W-Ir-B alloy. Region A: IrW phase; Region B: W_2_B phase. (B) W 4f deconvoluted core-level peaks of various materials after OER test. (C) Activity degradations for three catalysts at the current densities of 10 and 100 mA cm^−2^_geo_, respectively. (D) OER polarization curves of W-Ir-B alloy in 0.5 M H_2_SO_4_ electrolyte. (E) Aberration-corrected HAADF-STEM image of Ir-MnO_2_(160)-CC. (F) Schematic diagram of the three existing forms of Ir species.

Metal-support interaction effect refers to the electronic interaction between the active metal center of catalyst and its support material.^121^^,^^122^^,^^123^ For example, as the state-of-the-art OER electrocatalysts in acidic media, Ir-based materials possess balanced electrochemical activity and stability but inevitably suffer from dissolution, especially under high potential conditions. In this sense, selecting suitable supports and constructing strong interactions between them and Ir species may be an effective strategy for solving dissolution problem, and from the engineering perspective, this is crucial for PEM electrolyzer.^124^^,^^125^^,^^126^ Strasser group confirmed that metal-support interaction effect made an important contribution to the corrosion resistance stability of Ir-based electrocatalysts in acidic water splitting.^121^ Electronic interaction between catalyst and support can effectively slow down the rate and extent of Ir oxidation, as well as the dissolution of active NPs at anodic oxidation potentials. It is well known that the rate and extent of dissolution for anodic metals is related to their oxidizability.^127^ As more positive oxidation states appeared, Ir atoms suffered from increased dissolution. Conversely, a stable lower average Ir oxidation state would slow down anodic metal dissolution.^127^ When comparing the interaction of carbon and ATO supports with catalysts, it was found that the carbon support was oxidized at relatively low anodic potential. In contrast, ATO support significantly improved the corrosion dissolution stability of IrO_x_ particles due to interfacial electron transfer to the active Ir centers caused by interfacial interactions between the support and catalyst.^121^ Specifically, metal-support interaction effect made the voltammetric conversion of metallic Ir to IrO_x_ more difficult, which reduced the effective layer thickness of IrO_x_ and led to a continuous decrease in the Ir oxidation state.^121^ In an attempt to investigate the load-stabilizing effect of the support material, a biphasic W-Ir-B alloy possessing situ-formed IrW nanochannels with ∼200 nm depth for acidic water oxidation was synthesized by Li and colleagues (Figure 9A).^126^ XPS results revealed that the metallic state W^0^ is oxidized (WO_3_) during the OER process (Figure 9B). Thanks to the adsorption of excess O atoms by the hypervalent oxidation of W during the electrocatalytic process, the surface Ir atoms were prevented from accumulating more O atoms to form soluble higher valent species (Figure 9C). Thus, the catalyst-support interaction not only changed the charge distribution of surface Ir and O atoms (i.e., (IrO_2_)_n_ clusters) and effectively limited the cluster size to 4 (n ≤ 4), but also hindered the agglomeration of surface Ir. What’s more, the thin active IrO_2_ cluster layer retained on the surface enabled the catalyst to achieve super high activity and stability toward acidic OER (Figure 9D).^126^

Very recently, by introducing acid-resistant MnO_2_ nanowires as substrate and restricting Ir species to CC, Ir-MnO_2_(160)-CC catalyst was constructed by Xu and coworkers, which showed excellent activity and stability during the acidic OER process (Figure 9E).^128^ Specifically, the high-valence Mn coming from the modulation of potassium permanganate as oxidant can promote the redox reaction between Mn ions and IrCl_6_^2−^, resulting in the formation of Mn-O-Ir coordination structure (Figure 9F). Such coordination structure provided more anchor sites for the extra Ir ions to form strongly anchored Ir atom array on MnO_2_. It was evident that the unsaturated coordination environment of Ir can be effectively tailored by the charge transfer between Mn and O-Ir coordination structures. On the contrary, when reacting with low-valence Mn, Ir tended to aggregate into IrO_x_ NPs rather than forming Mn-O-Ir coordination, resulting in poor OER activity and stability.^128^ In addition, DFT calculations further revealed that anchoring atomic Ir to MnO_2_ can optimize the adsorption ability of HOO∗ intermediates for superior activity.

Active Ru-based catalysts tend to be overoxidized to form soluble high valent Ru ions (such as RuO_4_) under high potentials during acidic OER process, generally accompanied by the collapse of catalytic structure and intensified leaching of active Ru species, resulting in a decrease in stability. In addition to tuning the electronic structure of active metal center to improve catalytic activity, the metal-support interaction effect provides a constant supply of electrons to prevent overoxidation and corrosion in acidic medium.^129^^,^^130^ For example, by embedding Ru single atoms into acid-resistant N-C support, Cao et al. designed Ru-N-C catalyst toward acidic OER.^131^ A slight shrinkage in Ru-N bonds was observed under OER operating condition compared to that at open potential, which could further anchor Ru atom on the N-C support, thereby preventing potentially undesired dissolution and offering a superior stability of catalyst during the continuous electrolysis. Specifically, SR-FTIR and XAFS analyses revealed that O-Ru_1_-N_4_ site was formed by single oxygen pre-adsorption onto Ru_1_-N_4_ site at working potential, while Ru atoms transferred electrons to nearby N atoms and adsorbed O atoms through orbital hybridization. This optimized the binding energy of oxidation intermediates, thereby improving the catalytic activity during the acidic OER process. More recently, Sun et al. fabricated RuO_2_–WC NPs by the salt-templated method, showing the excellent activity of 347 mV at 10 mA cm^−2^ toward acidic OER.^129^ The DFT analysis demonstrated that RuO_2_ modified by WC support had a stronger electron transfer ability than RuO_2_ at high potentials. Such electronic structure modulation under catalyst-support interaction could optimize the adsorption energy of the active sites, lower the reaction barrier of RDS, and accelerate the kinetics of the OER process. In addition, the electrons transferred from WC to RuO_2_ during the OER process can protect the Ru active sites from over-oxidation, thus improving the stability of the catalyst.

Metal support interaction effect effectively tunes the unsaturated coordination environment of active species through charge transfer between supporting material and catalytic active center, which provides a solid foundation for the formation of strongly anchored catalytic arrays of metal centers on the supported substrate. Moreover, this strategy is expected to become an important means to effectively solve the problem of metal dissolution.

### Atomic dispersed engineering

**Figure 10:**
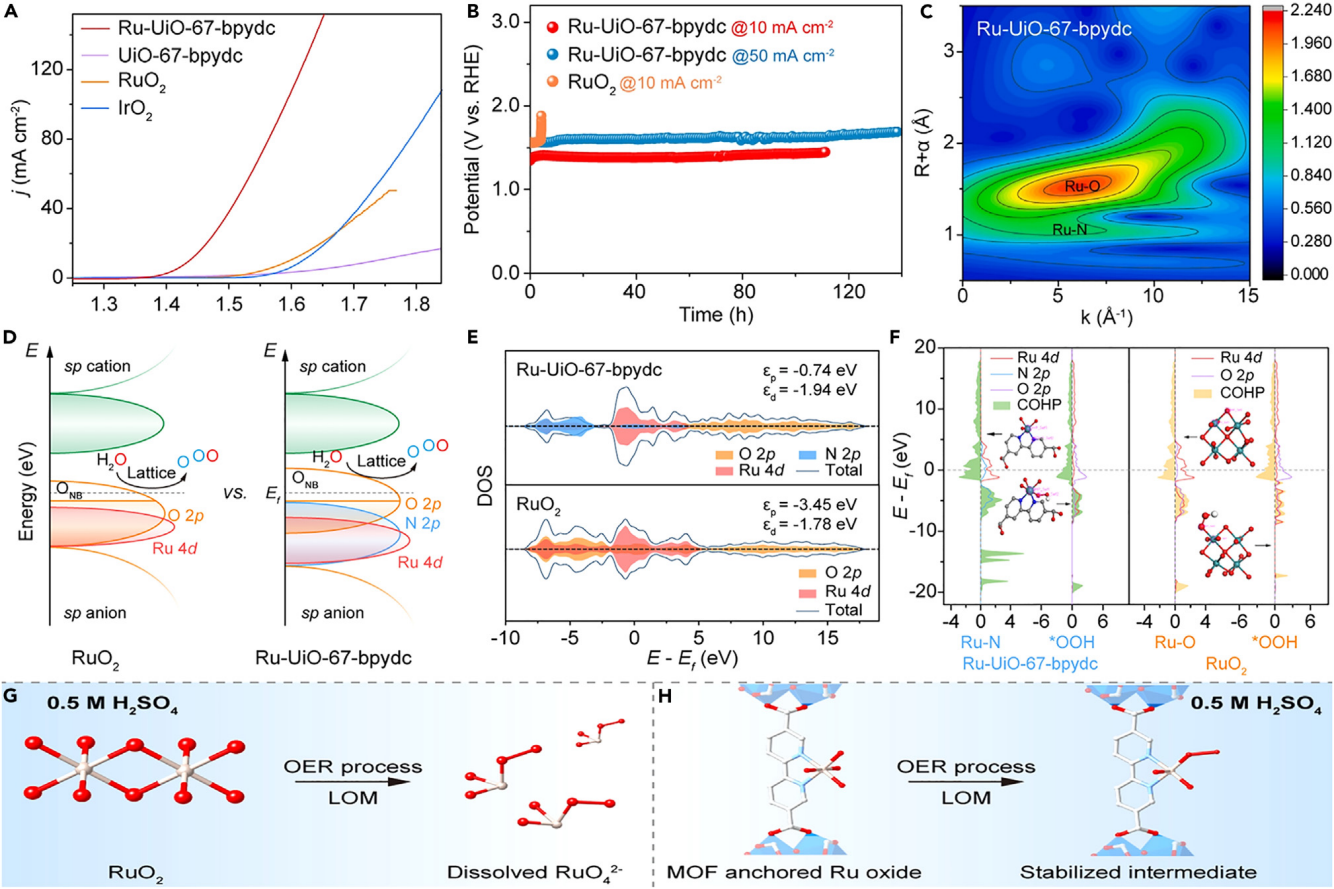
The illustration and characterization of Ru-UiO-67-bpydc catalyst during the acidic OER process Reproduced with permission from (Yao et al., 2023). Copyright @ 2023 Elsevier Inc. (A) Curves of various materials in oxygen-saturated 0.5 M H_2_SO_4_ electrolyte. (B) Chronopotentiometric curves of Ru-UiO-67-bpydc and RuO_2_. (C) Wavelet transform of Ru K-edge EXAFS data of Ru-UiO-67-bpydc. (D) Schematic molecular orbital energy diagram for Ru-UiO-67-bpydc and RuO_2_ toward the acidic OER. (E) DOS plots of Ru 4d, N2p, and O 2p states in Ru-UiO-67-bpydc; and DOS plots of Ru 4d and O 2p states in RuO_2_. (F) –COHP of Ru–N and Ru–OOH bond in Ru-UiO-67-bpydc and –COHP of Ru–O and Ru–OOH in RuO_2_. (G) The transformation of RuO_2_ to RuO_4_^−2^ during the acidic OER process by the LOM pathway. (H) The stability of the Ru intermediate in MOF-anchored Ru oxide during the acidic OER by LOM pathway.

Owing to the high atom utilization and catalytic efficiency, adjustable coordination environments, and versatile electronic structure, atomic dispersed metal catalysts have attracted more attention in electrocatalysis field.^39^^,^^132^^,^^133^^,^^134^^,^^135^ For example, by immobilizing atomically dispersed V active sites on N-doped multichannel carbon nanofibers employing electrostatic spinning, pyrolysis and chemical leaching procedures, V@NMCNFs electrocatalyst was synthesized toward efficient water oxidation in acidic environment, achieving atomic-level dispersion of V atoms and formation of open mesoporous channel structures.^136^ The asymmetric penta-coordinated V-O_2_N_3_ configuration with axial V-O coordination, evidenced by experiment and theoretical calculation results, can effectively optimize the electronic state distribution of the V-site, accelerate the charge transfer, and lower the free energy barrier of RDS, thereby significantly enhancing the intrinsic activity of catalytic material. The catalyst required only 196 mV overpotential to deliver a current density of 10 mA cm^−2^. Moreover, the unique cavity structure and open multichannel of CNFs ensured the accessibility of active sites, which greatly promoted the reaction kinetics. Benefiting from N-doping in the carbon matrix, the surface electron distribution of carbon was optimized, and more catalytic active sites toward OER were created. Particularly, due to the close binding of V single atoms in N-doped CNFs, the aggregation and reconstruction of active sites were effectively prevented during the acidic catalytic process, significantly promoting the stability of catalyst.^136^ Ru-UiO-67-bpydc was fabricated by anchoring atomically dispersed Ru oxide to UiO-67-bpydc using a simple hydrothermal method, which showed excellent performance and long-term stability in acidic OER (Figures 10A–10C).^137^ The oxygen evolution of this catalyst in acidic media followed LOM pathway rather than AEM process, as confirmed by isotope labeling, *in situ* Raman, and ^18^O-labeled differential electrochemical mass spectrometry (DEMS) analyses. Meanwhile, DFT calculations indicated that the strong Ru-N bonds between Ru oxide and MOF activated the lattice oxygen-induced LOM pathway by forming electron holes in the p-band of oxygen ligands when significantly lowering the d-band center of Ru and upshifting the p-band center of O (Figures 10D and 10E). In addition, the crystal orbital Hamilton population (COHP) demonstrated that the Ru-N bond in ∗VO-RuO_4_^−2^-UiO-67-bpydc was more stable than Ru-O in ∗VO-RuO_4_^−2^-RuO_2_ under Fermi level (Figure 10F), stabilizing the soluble oxygen-vacancy intermediates (∗VO-RuO_4_^−2^), and thereby enhancing the stability of acidic OER (Figures 10G and 10H).^137^

As mentioned above, in addition to facilitating the full exposure of active sites and therefore faster OER kinetics, atomic dispersion engineering can effectively tailor the electronic state distribution of the catalytic centers, accelerates charge transfer, and lowers the energy barriers of reaction intermediates, significantly boosting the intrinsic activity of electrocatalysts. In particular, the catalytic materials with atomic dispersion level should effectively prevent the aggregation and reconstruction during acidic OER process, so as to avoid possible adverse influence on the stability of catalysts.

### Spin electron reconfiguration

Recently, spin reconfiguration induced by atomic disorder hybridization was identified as a feasible strategy for developing novel electrocatalysts with high activity and stability.^138^^,^^139^^,^^140^ In theory, the intrinsic physical properties of the material may be altered under room temperature thermal disturbance, triggering atomic disordered hybridization and therefore a decrease in crystal symmetry.^139^ More importantly, this can open up some nondegenerate orbits, leading to the spin electron reconfiguration. For Ru-based catalysts, the overoxidation of Ru sites at high potentials during acidic OER leads to the generation of soluble high-valent Ru derivatives, which are the fundamental reason for the collapse of crystal structure and poor stability. According to Liu group’s speculation, if the bonding strength between the Ru atoms and the coordinated anions was greater than the redox H_2_O/O_2_ energy through electronic structural modulation, it can effectively hinder the overoxidation of Ru-based catalysts toward acidic OER process.^139^ Inspired of this, they disclosed the spin electron reconfiguration of Bi_x_Er_2-x_Ru_2_O_7_ catalyst driven by symmetry breaking, which played a critical role in determining the catalytic activity during the acidic OER process. The introduction of the temperature-sensitive Er atom partially replaced the Bi site, which locally reduced the structural disorder of the RuO_6_ coordination polyhedron, resulting in the formation of the half-disordered hybrid Bi_x_Er_2-x_Ru_2_O_7_. According to the octahedral crystal field theory, the filling of t_2g_ and e_g_, orbitals of metal ions was crucial for their exchange interaction. When the magnetic ions bond with the coordinate anions, the metal sites with empty t_2g_ orbitals were prone to accept the valence electrons from conjugated O to form metal-O bonds. Free energy calculations of the OER pathway with four electrochemical steps showed that the formation of Ru-∗OOH was the rate-determining step. Based on the lowest energy principle, the half-disordered hybridization of Bi_x_Er_2-x_Ru_2_O_7_ was more conducive to improving the catalytic activity, but the Ru active sites were easier to catalyze OER reaction. When HOO∗ was adsorbed onto Ru sites, the unpaired O-2p orbitals hybridize more readily with the Ru-4d orbitals, and the electrons in Ru-4d orbitals were partially transferred to the unfilled O-2p orbitals, evidenced by COHP analysis. Ultimately, a long continuous OER electrocatalysis for over 100 h accompanied with a superior activity of ∼0.18 V at 10 mA cm^−2^ in acidic electrolyte was offered by Bi_x_Er_2-x_Ru_2_O_7_. Similar spin reconfiguration strategy was observed in IrMnOF@Ir catalyst, where Ir cations were deposited onto the F-doped MnO_2_ surface to form Ir clusters.^140^ In particular, the bonding strength between Ir sites and coordinated F anions was stronger than the redox H_2_O/O_2_ energy, which was beneficial for improving the stability of catalyst. When coupled DFT with Brader charge analysis, it was found that the adsorbed HOO∗ and neighboring Ir clusters or atomic chains can participate in orbital hybridization and charge transfer simultaneously, triggering the electron reconfiguration and therefore boosting the catalytic activity.

### Others

**Figure 11:**
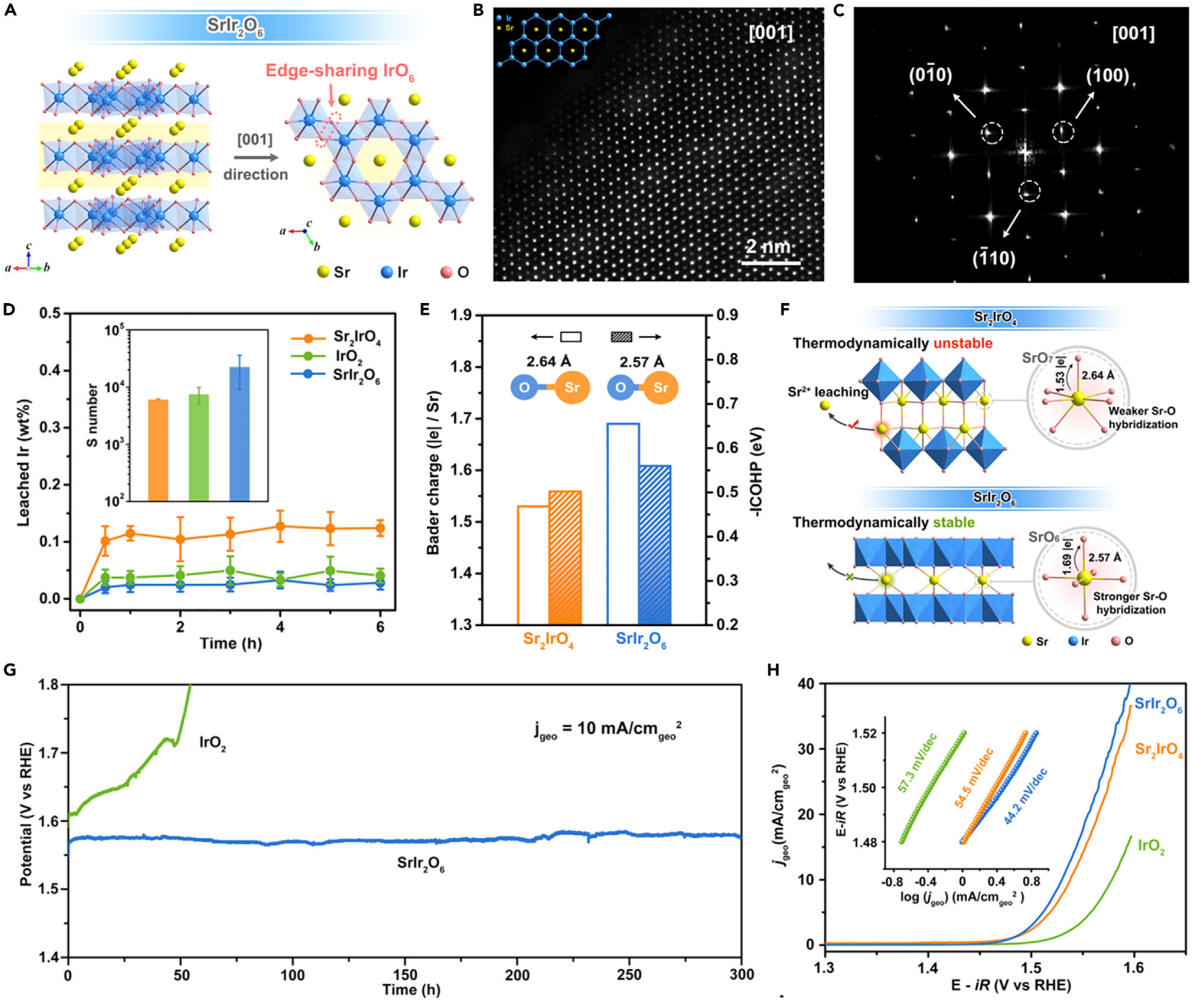
The illustration and characterization of SrIr_2_O_6_ during the acidic OER process Reproduced with permission from (Wang et al., 2023). Copyright @ 2023 American Chemical Society. (A) Crystal structures of crystal structures of SrIr_2_O_6_. (B) HAADF-STEM image and simulation structure model (inset) of SrIr_2_O_6_. (C) Selected area electron diffraction pattern of SrIr_2_O_6_. (D) Leached Ir amounts (wt %) from SrIr_2_O_6_, Sr_2_IrO_4_, and IrO_2_ during 6 h OER process, with the calculated S-numbers (inset). (E) Sr−O bond length, the integrated −ICOHP values of the Sr−O bond, and the Bader charge analysis of SrIr_2_O_6_ and Sr_2_IrO_4_. (F) Schem of the key factors controlling the structural stability of SrIr_2_O_6_ and Sr_2_IrO_4_. (G) Chronopotentiometric curves of SrIr_2_O_6_ and IrO_2_ at 10 mA cm_geo_^−2^ without iR-compensations. (H) LSV polarization curves and the corresponding Tafel slopes (inset) for OER of SrIr_2_O_6_, Sr_2_IrO_4_, and IrO_2_ in 0.1 M HClO_4_.

In addition to above strategies, other efforts have been reported to effectively modulate the electronic structure of electrocatalysts toward efficient acidic OER, such as morphology control,^141^^,^^142^^,^^143^^,^^144^^,^^145^^,^^146^ amorphization engineering,^147^^,^^148^^,^^149^^,^^150^^,^^151^ and crystal phase modulation.^120^^,^^152^^,^^153^ For example, the Ir-rich shell of core-shell IrRu_x_@Ir electrocatalyst can effectively protect the Ru atoms of IrRu_x_ core from being dissolved, ensuring the structural stability of catalytic material during the acidic OER process.^145^ When coupled with XPS analysis, the fact of strong electronic interaction between the shell and core was confirmed. This induced the electron transfer from Ir to IrRu_x_, resulting in the increased oxidation state of the surface Ir sites, which promoted the performance of catalyst. Ultimately, the prepared IrRu_x_@Ir electrocatalyst harvested outstanding OER activity, requiring only 288 mV overpotential to drive a current density of 10 mA cm^−2^ in acidic medium. The membrane electrode assembly coupled with anodic IrRu_x_@Ir exhibited superior stability for 400 h at 50°C and 1 A cm^−2^ current density. Honeycomb layered strontium iridate (SrIr_2_O_6_) was successfully prepared as an efficient and stable acidic OER electrocatalyst by Zou and colleagues (Figures 11A–11C).^154^ Its edges acted as the active sites to enhance the adsorption of OER intermediates via AEM pathway. The dissolution experiments showed that the layered SrIr_2_O_6_ structure has only ∼0.4% Sr and 0.03% Ir leaching in acidic solution (Figure 11D), indicating the exceptional structural stability of catalytic material. This was attributed to the short Sr-O bond and the strong electron transfer from Sr to O in SrIr_2_O_6_, as well as the strong edge-sharing [IrO_6_] connectivity and the weak Ir-O covalency (Figures 11E and 11F). As a result, SrIr_2_O_6_ avoided the surface amorphization during the acidic OER process and maintained excellent catalytic activity for more than 300 h (Figures 11G and 11H).^154^

Investigating the origin of amorphization in the surface layer of electrocatalytic materials, Yan group found that acidic anions played a key role in the long-term catalytic stability of Ca_2-x_IrO_4_ nanocrystals.^155^ In contrast to the almost unchanged Ca coordination environment in the HClO_4_ electrolyte, a small amount of CaSO_4_ molecules were observed to be adsorbed on the surface of Ca_2-x_IrO_4_ in H_2_SO_4_ electrolyte, suggesting that the obvious amorphization occurred on the surface of catalytic material. The strong binding strength of SO_4_^−2^ and CaSO_4_ on Ca_2-x_IrO_4_ promoted the surface amorphization of Ca_2-x_IrO_4_, and the formation of both crystalline and amorphous IrO_x_ layers on the surface were confirmed to be excellent catalytic active species, which are responsible for the enhancement of OER activity. However, the SO_4_^−2^ in H_2_SO_4_ electrolyte significantly destabilized the Ca_2-x_IrO_4_ surface, leading to a decrease in the electrocatalytic stability of the acidic OER.^155^ In 2019, Zhao et al. synthesized Ru octahedral nanocrystals following fcc filling by crystalline phase modulation, whose specific activity for acidic OER was shown to be 4.4 times higher than that of conventional hexagonal close-packed (hcp) lattice typical of bulk Ru.^156^ Integration of bulk and nanoparticle benefits into "macro-nano" catalysts through crystalline phase modulation was confirmed to be a powerful strategy to address the balance between activity and stability by Fan et al.^120^ The prepared 2D fcc-Ru_3_Ir nanocrystals exhibited strong acidic OER properties.^120^

### Conclusions and perspectives

PEM water electrolyzers offer a promising pathway for producing sustainable hydrogen energy. However, under harsh acid corrosion conditions, the sluggish kinetic OER hinders the overall efficiency of the water electrolyzers. Efficient and durable electrocatalysts are necessary to reduce the kinetic barrier of OER. To improve the efficiency of acidic water oxidation, various advanced electrocatalysts represented by Ir- and Ru-based nanostructures have been successively designed and synthesized. It has been demonstrated that the electronic structure of catalytic materials is closely related to their OER activity and stability and can be achieved through various pathways (Table 1). Specifically, optimizing composition through defect and doping engineering, modulating coordination environment through alloy engineering, performing atomic arrangement through morphology and interface control, as well as surface reconstruction and constructing metal-support interactions, have been demonstrated to promote OER through electronic structure regulation. Although extensive progress has been made in improving the performance of acidic OER, research on many fundamental aspects such as accurately identifying the true active sites and stable species, determining dynamic structure-durability relationship, and establishing benchmark system for catalytic performance evaluation is still on its exploring period. Many challenges remain for the future in developing electrocatalysts with remarkable activity and superior durability toward acidic OER.

1. Breaking the scaling relationship and enhancing the application of non-precious transition metals. Currently, the development of acidic OER electrocatalysts is still heavily relies on scarce precious metals such as iridium and ruthenium, which is a bottleneck restricting the large-scale application of PEM electrolyzers. Consequently, future research should focus on reducing the use of precious metals and developing earth-abundant alternatives to improve the efficiency of acidic OER catalysts. The "volcano diagram" provides a powerful and low-cost method for the rapid screening of electrocatalysts. However, as is well known, even the most promising Ir-based catalysts still require substantial overpotentials during acidic electrocatalysis. Obviously, such scaling relationship hinders the development of electrocatalysts. In this sense, breaking this scaling law to reduce overpotential and improve catalytic efficiency is an important direction for the development of catalysts in the future.
2. Precise identification of active sites and stable phases as well as the determination of reaction mechanisms play a crucial role in the development of acidic OER electrocatalysts. The catalytic activity and stability between different catalysts cannot be compared and explained by any one mechanism. Due to oxidation potential and acid corrosion, the surface of OER catalysts tends to undergo dynamic change during the catalytic process, posing an obstacle to the identification of true active sites and the determination of the reaction mechanism. Therefore, in addition to investigating the structural evolution laws of reaction intermediates or catalysts during electrocatalysis with the help of *in situ* techniques, more powerful simulation techniques should be developed to elucidate the OER reaction mechanisms under different conditions. Through the effective combination of these two approaches, the active sites and reaction mechanisms of OER electrocatalysts are elaborated, providing important information for the design of robust electrocatalytic materials.
3. The important role of electronic structure modulation in improving catalyst stability should be fully utilized. The dissolution issue can be addressed by changing the coordination environment of metal atoms. For example, for Ru-based catalysts, selecting a metal with weak oxygen bond bonding and embedding atomic Ru into this metal-rich coordination environment can improve the solubility resistance of Ru and inhibit its dissolution. Alternatively, triggering the spin-electronic reconfiguration of catalysts driven by symmetry breaking makes the bonding strength between Ru atoms and ligand anions larger than the redox energy of H_2_O/O_2_, thus effectively preventing the overoxidation of Ru-based catalysts.
4. The stability of acidic OER catalysts remains a great challenge. Under industrial condition, a satisfactory OER electrocatalyst should be able to operate stably at high current densities greater than 2 A cm^−2^ and low overpotentials less than 500 mV.^81^ However, even the most promising Ir-based OER catalysts slowly dissolve under acidic electrocatalysis, leading to reduced performance and Ir loss. Consequently, tracking the changes in composition, valence, coordination environment, and conductivity of catalytical active species through *in situ* characterization techniques is necessary for the comprehensive stability evaluation of electrocatalysts. Coupled with theoretical calculations to further elucidate the electrochemical degradation mechanism of catalytical materials is crucial to address the stability issue.
5. Reasonable establishment of a rigorous benchmark evaluation system for electrocatalyst performance. Compared with the industrial-scale application, the traditional laboratory-scale test has bias in evaluating the performance of catalysts due to the differences in catalyst size, mass loading, matrix effect, and test conditions. More importantly, the OER activity and stability trends of catalysts in PEM electrolyzers may differ from the test results of conventional methods, such as three-electrode test systems. Therefore, to quantitatively compare the performance of different catalysts, a rigorous and reasonable benchmark evaluation method for catalytic performance should be constructed, comprehensively considering parameters such as overpotential, exchange current density, Tafel slope, turnover frequency, Faraday efficiency, long-term stability, and electrochemically active surface area during the catalytic process. Furthermore, considering the future industrial practicality of catalysts, the performance evaluation of synthesized catalytic materials in PEM electrolyzers will be more objective and persuasive. To avoid invalid or misleading comparisons, establishing a standard assembly method for PEM electrolyzers is a better choice.

**Table 1:** The OER activity, corresponding reaction mechanisms and modification strategies of some representative electrocatalysts in acidic media

Catalyst	Electrolyte	Overpotential (mV vs. RHE @10 mA cm^−2^)	Reaction mechanism	Modification strategy	Reference
Ru/MnO_2_	0.1 M HClO_4_	161	OPM	Metal-support interaction effect	Lin et al.^64^
IrCoNi hollow nanocrystal	0.1 M HClO_4_	303	AEM	Alloy engineering	Feng et al.^88^
Ru–Ir alloy NPs	0.5 M H_2_SO_4_	292	AEM	Alloy engineering	Guo et al.^89^
ZnNiCoIrMn	0.1 M HClO_4_	237	AEM	Alloy engineering	Kwon et al.^95^
Nb–RuO_2_ NPs	0.1 M HClO_4_	204	AEM	Heteroatom doping	Liu et al.^101^
S-doped M-SrIrO_3_	0.5 M H_2_SO_4_	228	AEM	Heteroatom doping	You et al.^104^
B-doped RuO_2_	0.5 M H_2_SO_4_	200	AEM	Heteroatom doping	Liu et al.^105^
IrNi@IrOx core-shell NPs	0.5 M H_2_SO_4_	300	LOM	Defect engineering	Nong et al.^60^
Rh–RuO_2_/G	0.5 M H_2_SO_4_	161	AEM	Defect engineering	Wang et al.^108^
TiMnCoCN	0.5 M H_2_SO_4_	143	AEM	Defect engineering	Zheng et al.^110^
Zn-doped RuO_2_	0.5 M H_2_SO_4_	190	AEM	Defect engineering	Zhou et al.^112^
IrO_x_/SrIrO_3_	0.5 M H_2_SO_4_	270	AEM	Surface reconstruction	Seitz et al.^78^
RuMn alloy	0.5 M H_2_SO_4_	237	–	Surface reconstruction	An et al.^118^
2D fcc-Ru_3_Ir	0.5 M H_2_SO_4_	190	AEM	Surface reconstruction	Fan et al.^120^
W-Ir-B alloy	0.5 M H_2_SO_4_	∼291 mV	–	Metal-support interaction effect	Li et al.^126^
Ir-MnO_2_(160)-CC	0.5 M H_2_SO_4_	181	AEM	Metal-support interaction effect	Weng et al.^128^
RuO_2_–WC NPs	0.5 M H_2_SO_4_	347	AEM	Metal-support interaction effect	Sun et al.^129^
Ru-N-C	0.5 M H_2_SO_4_	267	AEM	Metal-support interaction effect	Cao et al.^131^
Ir_1_-Co_3_O_4_-NS	0.5 M H_2_SO_4_	226	AEM	Atomic dispersed engineering	Liu et al.^135^
V@NMCNFs	0.5 M H_2_SO_4_	196	AEM	Atomic dispersed engineering	Li et al.^136^
Ru-UiO-67-bpydc	0.5 M H_2_SO_4_	200	LOM	Atomic dispersed engineering	Yao et al.^137^
Bi_x_Er_2-x_Ru_2_O_7_	0.1 M HClO_4_	∼180	AEM	Spin electron reconfiguration	Zhou et al.^139^
IrMnOF@Ir	0.1 M HClO_4_	170	AEM	Spin electron reconfiguration	Xu et al.^140^
Layered iridate nanosheets	0.1 M HClO_4_	300	AEM	morphology control	Chen et al.^141^
ruthenate nanosheets	0.1 M HClO_4_	255	AEM	morphology control	Laha et al.^143^
α-RuTe_2_ porous nanorods	0.5 M H_2_SO_4_	290	AEM	amorphization engineering	Wang et al.^147^
IrO_2_@LnIr_1-n_O_x_(OH)_y_	0.1 M HClO_4_	295	AEM & LOM	amorphization engineering	Ma et al.^148^
3R-IrO_2_	0.1 M HClO_4_	188	AEM	crystal phase modulation	Fan et al.^152^
SrIr_2_O_6_	0.1 M HClO_4_	303	LOM	crystal phase modulation	Wang et al.^154^

### Limitations of the study

This review only focuses on electrocatalysts with enhanced catalytic performance by tuning the electronic structure toward acidic OER. Electronic structure tuning strategies can also boost the activity of catalysts under alkaline conditions, which may show different results from what we present in this review.
